# Phytochemical Characterization of Five Edible Purple-Reddish Vegetables: Anthocyanins, Flavonoids, and Phenolic Acid Derivatives

**DOI:** 10.3390/molecules24081536

**Published:** 2019-04-18

**Authors:** Alexandra D. Frond, Cristian I. Iuhas, Ioana Stirbu, Loredana Leopold, Sonia Socaci, Stǎnilǎ Andreea, Huseyin Ayvaz, Socaciu Andreea, Socaciu Mihai, Zorița Diaconeasa, Socaciu Carmen

**Affiliations:** 1 Faculty of Food Science and Technology, University of Agricultural Sciences and Veterinary Medicine Cluj-Napoca, 3-5 Calea Manastur, Cluj-Napoca 400372, Romania; frondalexandradenisa@gmail.com (A.D.F.); ioanaalexandrastirbu@gmail.com (I.S.); loredana.leopold@usamvcluj.ro (L.L.); sonia.socaci@usamvcluj.ro (S.S.); andreea.stanila@usamvcluj.ro (S.A.); carmen.socaciu@usamvcluj.ro (S.C.); 2 Faculty of Medicine, Iuliu Hatieganu University of Medicine and Pharmacy, Cluj-Napoca 400372, Romania; iuhascristianioan@yahoo.co.uk (C.I.I.); medisyn@yahoo.com (S.A.); socacium@yahoo.com (S.M.); 3 Faculty of Engineering, Canakkale Onsekiz Mart University, Canakkale 17020, Turkey; huseyinayvaz@comu.edu.tr

**Keywords:** polyphenols, antioxidants, anthocyanins, phenolic acids, flavonoids, HPLC

## Abstract

Vegetables comprise a significant portion of our daily diet with their high content in nutrients including fiber, vitamins, minerals, as well as phenolic compounds. Vegetable consumption has been shown to be positively associated with the prevention of several degenerative diseases thanks to their bioactive compounds. Accordingly, five selected vegetables, namely, red chicory, red onion, eggplant, purple sweet potato, and black carrot were thoroughly assessed for their phenolic content in this study. For this purpose, the total phenolic and flavonoid content of these five vegetables and their antioxidant activities that are based on three common methods ABTS radical cation decolorization assay (ABTS), Cupric Ion Reducing Antioxidant Capacity (CUPRAC), and DPPH scavenging activity assay were determined. Additionally, HPLC-PDA/Electrospray ionization coupled with mass spectrometry (HPLC-PDA/-ESI^+^-MS)-based identification and quantification of the members belonging to polyphenols present in each vegetable were determined. Statistical correlations between antioxidant activities and the specific type of phenolic compounds, such as anthocyanins, flavonoids, anthocyanins, and phenolic acids were further elucidated. Phenolic acids (chlorogenic and syringic acids) were found to be the most abundant compounds that are present in all samples. Among the anthocyanins, cyaniding derivatives were present in all vegetables. In terms of their antioxidant activities, the analyzed vegetables were ranked as red chicory > purple sweet potato > black carrot > eggplant > red onion, in descending order. Superior antioxidant activities exhibited by red chicory and purple sweet potato were attributed to the high content of phenolic compounds, especially flavonols (quercetin-3,4-*O*-diglucoside) in red chicory and anthocyanins (peonidin-3-caffeoyl p-hydroxybenzoylsophoroside-5-glucoside) in purple sweet potato.

## 1. Introduction 

Polyphenols are secondary major metabolites that are found in plants with various structures, including lignin, tannins, phenolic acids, flavonoids, and numerous derivatives [1]. They are essential in various functions in plants and that are currently used in many food or pharmaceutical applications. Plant metabolites are produced for their own growth and reproductive purposes, but they are also essential for human health. Among these natural metabolites, polyphenols is a large class that is found in a wide variety of fruits, vegetables, seeds, flowers, beverages, and various foods. Among them, berries, vegetables, coffee, wine, or chocolate are common sources of polyphenols in the human diet [2]. 

**Figure 1:**
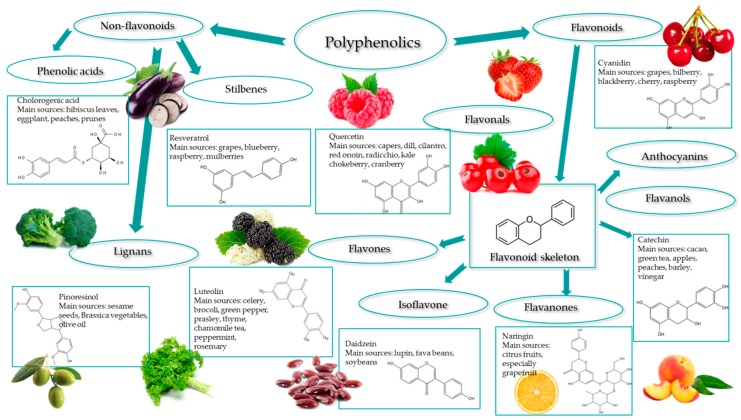
Polyphenols: classification and main sources (adapted from Goszcz, K. et al., 2015 [5]).

Regarding their chemical structure, phenolic compounds consist of minimum of one phenol group (aromatic ring). They can be sub-grouped as flavonoids and non-flavonoids, as illustrated in Figure 1. Phenolic acids, which are listed under the group of non-flavonoids, are hydroxyl derivatives of aromatic carboxylic acids that have a single phenolic ring and they can be either C1–C6 or C3–C6. Flavonoids contain two phenolic rings that are linked by a carbon bridge of an oxygenated heterocycle, having a C6–C3–C6 skeleton. Under the category of flavonoids, anthocyanins naturally occur as glycosides and they have one or more sugars attached to an aglycon nucleus (anthocyanidin), with the exception of 3-deoxyanthocyanins [3]. Initially, the interest for these compounds was due to the bitterness, astringency, color, and odor that they exhibited. However, they were later recognized for their nutritional value and associated potential health benefits [4].

Various nutritional, clinical, and epidemiological studies have shown that polyphenols strengthen health, prevent various neurodegenerative diseases, including cancer and metabolic disorders [6]. There are also numerous studies [7,8] that attest to the potential benefits of polyphenols in cancer and diabetes, where both vegetables and fruits are recognized for their rich phytochemical content, such as blue maize and various berry fruits. The ability to suppress oxidation is the most evident benefit all these potential benefits to human health. The exposure of tissues to various aggressive factors causes inflammation, but also the release of reactive oxygen species (ROS), which may be beneficial in preventing the invasion of various pathogens. However, such a long response may lead to tissue damage and irreversible degradation in some molecules, such as DNA or proteins [9]. There is consistent evidence to support that polyphenols can prevent this oxidative damage and reduce inflammation [10]. Fruits, vegetables, cereals, teas, essential oils, various beverages, or food products that are derived from them contain natural antioxidants and chemopreventive agents, such as polyphenols, so a diet that is rich in polyphenols is highly advised. The recommended daily amount of polyphenols is 1177 mg for men and 1192 mg for women [11]. 

Antioxidant and anti-inflammatory activities, as well as other biological functions of polyphenols, were attributed to their chemical structure. The aromatic structure and the multitude of hydroxyl groups make these compounds good donors of electrons and hydrogen atoms, neutralizing free radicals and other ROS [3]. There is much evidence regarding the antioxidant and anti-inflammatory effect of polyphenols in vitro, but this effect is not always found in vivo. The inconsistency of in vitro and in vivo observations raises many questions, especially about their role in human health. Free or conjugated forms of polyphenols are absorbed in the upper gastrointestinal tract, but their bioavailability is low [12]. Phenolic compounds are metabolized before they can be transported to different tissues and organs through circulation, where they can exert their effects. Unabsorbed compounds can be metabolized or released by the microbiota of the colon, having a local anti-inflammatory or an indirect effect promoting the growth of probiotics [13]. 

When considering all of the great benefits of phenolic compounds, screening the possible sources of the them and discovering their individual members that are present in each plant of interest is a popular research area. Exploring new sources of polyphenols or deeper investigation of them may create new opportunities that are associated with global nutrition and food requirements [14]. It is critical to know the qualitative and quantitative distribution of the phenolic compounds, such as phenolic acids, flavonoids, anthocyanins in medicinal plants, fruits or vegetables, and their other related characteristics, including their antioxidant properties. Accordingly, this study targeted an inclusive understanding of phenolic composition of five selected red-fleshed vegetables that are commonly consumed in Europe: eggplant (*Solanum melongena*), red chicory (*Cichorium intybus*), red onion (*Allium cepa*), purple sweet potato (*Ipomoea batatas*), and black carrot (*Daucus carota cultivar Deep Purple*). Determining the total phenolic and flavonoid content of these five vegetables, their antioxidant activities that are based on three common methods, mass spectroscopic identification, and quantification of the members of these phenolic compounds present in each vegetable, including anthocyanins and statistical correlation between antioxidant activities and the specific type of phenolic compounds, such as anthocyanins, flavonoids, anthocyanins, and phenolic acids were aimed at this study. Although some of this information could be readily available in the literature, a more comprehensive investigation was targeted at this attempt. Additionally, to the best of our knowledge, the assessment of the impact that was exhibited by the specific type of phenolic compounds on the antioxidant activity was examined for the first time with this effort. 

## 2. Results and Discussions

### 2.1. Total Polyphenol and Flavonoid Contents

We first determined the phytochemical contents, such as total phenolics and flavonoids in extracts that were obtained from edible samples of red chicory, eggplant, red onion, black carrot, and purple sweet potato. As reported in Table 1, the extracts had high amounts of total phenolic and flavonoid compounds. The high levels of the total phenolic and flavonoid content found in our extracts are in a similar range as reported in the literature for different varieties of the same vegetables that were included in this study [6,15,16,17,18,19]. 

The total amount of phenolics found in our samples was in the range of 134.2–216.1 mg gallic acid equivalents (GAE)/100 g fresh weight (FW), where red chicory contained the highest amount, while eggplant had the lowest. Our results for red chicory (216.1 mg/100 g FW) and purple sweet potato (167.4/100 g FW) were similar to the ones that were reported by Abbas and Han [15,17]. 

Some variations were observed in comparison of the other three samples with the literature. In the eggplant, the concentration of phenolic compounds was 134.2 mg/100 g FW, where it was reported as 943.64 mg/100 g extract in the literature [16]. The concentration of phenolic compounds in red onion was 141.1 mg/100 g FW in our study, and the study with which we compared had 6.61 mg/g DW [6]. Additionally, the concentration that we obtained in black carrot was 167.4 mg/100 g FW, whereas 112.7 mg/g DW was reported in a previous study [18]. The differences in values are mostly due to the reporting the concentrations either in fresh or dry weight. We reported as fresh weight in our study, whereas Akanitapichat, Lisanti, and Toktas reported dry weight in the studies [6,16,18]. Additional factors that cause the variation in the results could be samples preparation, cultivation method, harvesting time, and such.

The range of total amount flavonoids that were found in our samples was between 101.79–22.14 mg rutin/100 g FW, where eggplant had the highest and black carrot had the lowest concentrations. Our results were compared with the available published data [15,16,17,18,19], where different variations were observed. The red onion that we analyzed contained a concentration of flavonoid of 72.24 mg/100 g FW, while a previous study reported a concentration of 110.1 mg/100 g FW. The results are relatively close in value, where both of the values were expressed as mg rutin equivalents/100 g FW [19]. In purple sweet potato, the flavonoid concentration that we obtained was 72.2 mg/100 g FW, and another study had a concentration of 65.7 mg/100 g powder [18]. The flavonoid concentration that was obtained in eggplant was 101.7 mg/100 g, while another study had a concentration of 1991.2 mg/100 g extract [16]. The red chicory concentration of flavonoids was 61.95 mg/100 g FW and the concentration given in a study in the literature was only 6.8 mg/100 g DW [15]. Black carrot contained a concentration of 22.1 mg/100 g FW, while another study expressed their results in 643.2 mg/100 g DW [18]. There are fewer studies in which the values are expressed as mg/g fresh weight. Although there was a lot of information regarding the total polyphenolics content of various samples in the literature, information on flavonoids was scarce.

### 2.2. Antioxidant Activity 

It is known that there is a correlation between antioxidant activity and phenolic content [20,21]. To investigate the antioxidant activity, three methods were tested in this study: ABTS, DPPH and Cupric Ion Reducing Antioxidant Capacity (CUPRAC) methods act in the same way and their working mechanism, namely single electron transfer (SET). The SET methods can detect the ability of a potential antioxidant compound to transfer an electron to reduce any other compound, including metals, carbonyls, and radicals [22]. 

**Figure 2:**
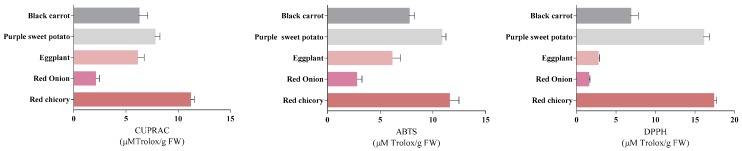
Antioxidant capacity of selected vegetables (values are expressed as µM Trolox/FW).

The DPPH method is based on measuring the reductive ability of antioxidants against DPPH, and this ability is measured as a decrease in the absorbance. The color is lost, following the reaction between the extract and the DPPH [23]. By the ABTS method, ABTS is oxidized by peroxyl radicals or other types of oxidants to an ABTS^+^ cation, which is highly colored [24]. CUPRAC is based on the reduction of Cu (II) to Cu (I) by the combined action of reducing agents in the extract. Absorbance measured is due to the concentration of the neocuproine-Cu (I) complex that formed following the reaction [25]. Figure 2 shows the antioxidant activity values that were obtained for the analyzed samples in our study. 

From the highest to the lowest, the order of the samples that were used in this study based on the antioxidant activity was red chicory > purple sweet potato > black carrot > eggplant > red onion. This order was constant for all three types of antioxidant assays used in this study, regardless of the assay used. Red chicory showed the highest activity in all three methods, where the values ranged between 11.2 ± 0.341–17.4 ± 0.346 µM/g FW. In the literature regarding the ABTS results, red chicory had a lower value than that determined in this study for red chicory (5.067 ± 35.6 µM/g FW) [26]. Red onion had the lowest antioxidant activity, where the values were between 1.6 ± 0.105 and 2.8 ± 0.469 µM/g FW. Following another study, the results that were obtained through ABTS and DPPH assay were higher and are in between 21.31 ± 0.41–22.90 ± 0.01 µM/g DW [6]. The other three samples exhibited antioxidant activity values that ranged between 7.8 ± 0.497–6.1 ± 0.604 µM/g FW for CUPRAC, 10.8 ± 0.420–7.7 ± 0.552 µM/g FW for ABTS, respectively, 16.1 ± 0.805–2.8 ± 0.140 µM/g FW for DPPH. The most significant difference between the literature and our antioxidant activity results was observed in purple sweet potato, presenting higher values than ours, such as: 7.25 ± 0.01 mM/100 g DW (ABTS) and 5.50 ± 0.01 mM/100 g DW (DPPH) and eggplant showing lower values than ours, such as 66.74 ± 4.60 µg/mL (DPPH) and 53.18 ± 0.71 µg/mL (ABTS) [16,27]. The explanation for those differences can be attributed to the extraction methods, solvents, and moreover the fact that the listed studies have used dried powdered samples. 

In order to verify the statistical correlation for antioxidant activity results, we used the Spearman’s rank correlation coefficient (or Spearman’s rho, Table 2). The Spearman’s rho values for each set of methods were as follows: rho CUPRAC, ABTS = 0,914; *p* < 0.001, rho CUPRAC, DPPH = 0.918; *p* < 0.001, rho ABTS, DPPH = 0.946; *p* < 0.001. The data indicated a strong correlation between the chosen methods. 

### 2.3. Liquid Chromatography/Electrospray Ionisation Mass Spectrometry (LC-ESI^+^-MS) Identification Analysis 

**Figure 3:**
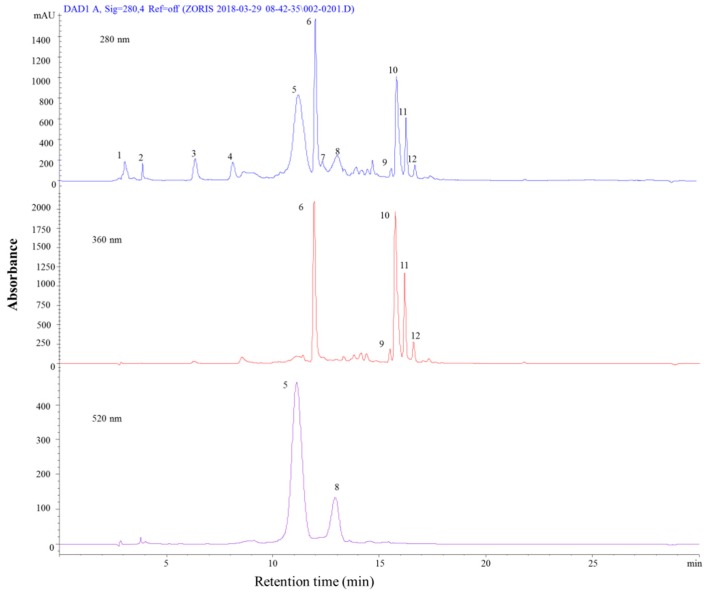
HPLC-PDA chromatograms of red chicory recorded at 280, 360 and 520 nm.

In this study, the chemical composition of phenolic compounds that were present in five different vegetables (red chicory, eggplant, black carrot, purple sweet potato, and red onion) was determined. Identification and peak assignment were primarily based on the comparison of mass spectrometric data with relevant literature [28,29,30]. For each sample, a representative chromatogram was recorded at 280, 360, and 520 nm. UV-Vis/MS spectra and chemical structure of each indetified compound are listed in Supplementary materials. Red chicory was found to contain a large number of phenolic compounds when compared to the other vegetables through the chromatographic analyses (Figure 3 and Table 3). Our results were in agreement with the literature studies regarding the identification of phenolic compounds in red chicory, as further explained [28,29,31,32]. 

The phenolic acids were represented by peaks 1–4 and 6, where the major peak 6 was assigned to chlorogenic acid (*m*/*z* = 355). The other phenolic acids were identified as hydroxybenzoic acid having [M − H]^+^ ion at *m*/*z* = 139 (peak 1), dihydroxybenzoic acid with ([M − H]^+^, *m*/*z* = 156) (peak 2), dihydroxy p-coumaric acid ([M − H]^+^, *m*/*z* = 167) (peak 3), and protocatechuic acid ([M − H]^+^, *m*/*z* = 155) (peak 4). Besides those that were identified in this study, some other compounds were previously identified and reported in a recent study [28], where they reported the presence of many derivatives of quinic acid, as well as malic acid and caffeic acid. Among them, three compounds were identical to the ones that were found in the present study.

The identified flavonoids were a total of five (peaks 7, 9–12), and they were represented by catechin ([M − H]^+^, *m*/*z* = 291) (peak 7), quercetin-3-*O*-rutinoside (Rutin) ([M − H]^+^, *m*/*z* = 611) (peak 9), quercetin-3-*O*-glucoside ([M − H]^+^, *m*/*z* = 465) (peak 11), quercetin-3-*O*-(6”-malonyl-glucoside) ([M − H]^+^, *m*/*z* = 551) (peak 12), and quercetin-3,4-*O*-diglucoside ([M − H]^+^, *m*/*z* = 625) (peak 10).

Among the ones determined in this study by Sahan [29], several other flavonoids were identified, such as kaempferol, isorhamnetin, and myricetin and their different derivatives. Peak 5 and 8 were identified as anthocyanins and both were cyanidin derivatives: cyanidin-3-*O*-glucoside with the molecular ion at [M − H]^+^, *m*/*z* = 449 and cyanidin-3-*O*-(6″-malonyl-glucoside) with the molecular ion at [M − H]^+^, *m*/*z* = 535. Besides the anthocyanins that were identified in this study, some other anthocyanins of delphinidin, malvidin, peonidin, and cyanidins, such as acylated ones (e.g. cyanidin-3-*O*-(6″-*O*-acetyl)-glucoside) were also reported in the literature [28].

The appearance of these differences with the literature could be linked to numerous factors, such as the type of the solvent used for extraction, the conditions in which the plant was cultivated, variety, climatic conditions, cultivation method harvesting period, and also analytical conditions (HPLC-MS).

**Figure 4:**
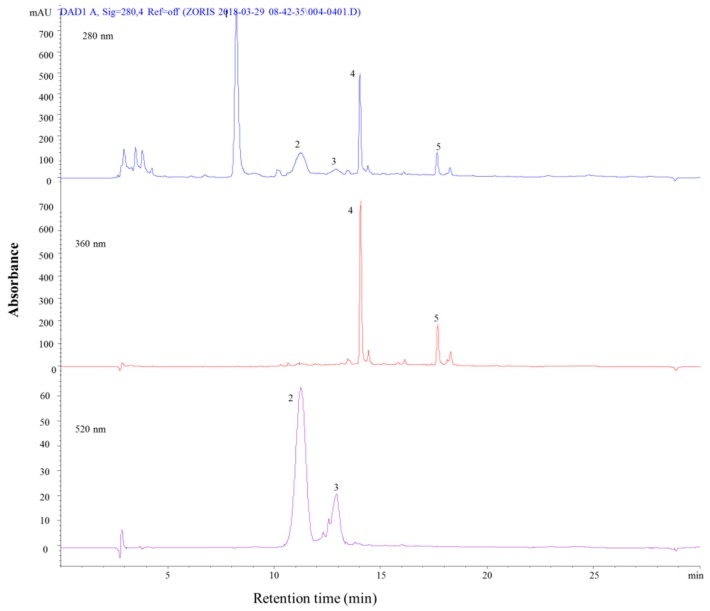
HPLC-PDA chromatograms of red onion recorded at 280, 360, and 520 nm.

Red onion was found to contain five compounds through the chromatographic analysis (Figure 4 and Table 4). The first compound identified was syringic acid, which is a phenolic acid (peak 1), with a molecular ion at [M − H]^+^, *m*/*z* = 198. The other two compounds identified (peak 2 and 3) were attributed to anthocyanins of glycosylated and ribiosylated cyanidins, respectively. 

It was also found that cyanidin-3-*O*-glucoside that was co-eluted with cyanidin-3-*O*-laminaribioside (peak 3) and cyanidin-3-(6″-malonyl-glucoside) co-eluted cyanidin-3-(6″-malonyl-laminaribioside) (peak 4). The last two identified compounds were flavonoids, with molecular ions of [M − H]^+^, *m*/*z* = 627 and [M − H]^+^, *m*/*z* = 465 at peak 4 and 5, which were identified as quercetin-3,4-*O*-diglucoside and quercetin-3-*O*-glucoside, respectively. These results for red onion followed the literature [33,34,35]. However, even though red onion is a popular vegetable, most of the literature was focused on anthocyanins and flavonoids, and little information was available regarding phenolic acids. Same derivatives of the anthocyanins and flavonoids identified in this research were also reported in other studies [34] cyanidin 3-(6″-malonyl-3″-glucosyl), cyanidin-3-(6″-malonyl-laminaribioside), and quercetin 4′-glucoside.

However, some other anthocyanins were identified and quantified in red onion, such as cyanidin 3-(3″-malonyl) glucoside, delphinidin-3,5-digalactoside, cyanidin 3-(3″-acetyl)glucoside, delphinidin 3,5-diglucoside, delphinidin-3-*O*-glucoside, and cyanidin 3-(malonyl)(acetyl) glucoside were also present in the literature [30]. 

**Figure 5:**
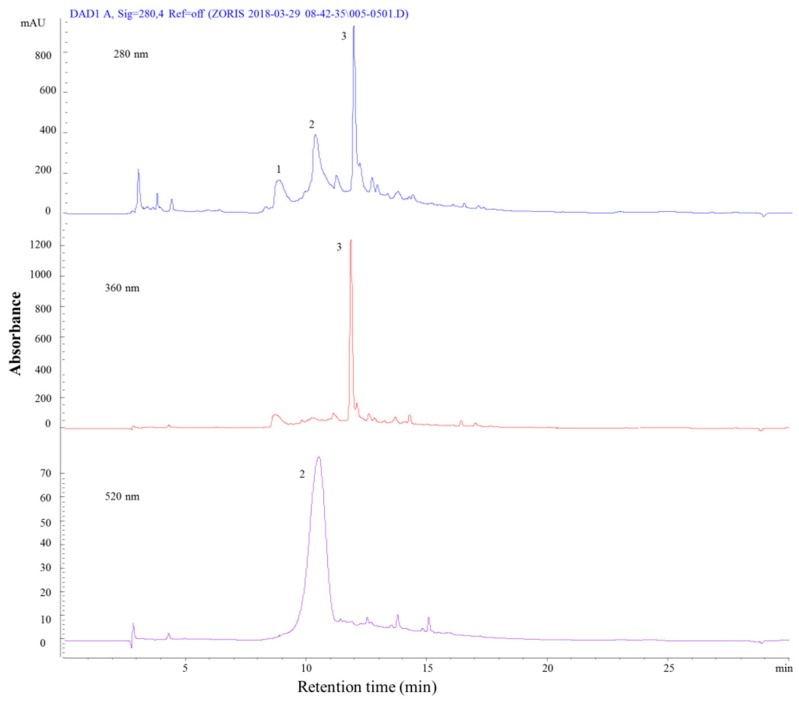
HPLC-PDA chromatograms of eggplant recorded at 280, 360 and 520 nm.

The eggplant had the fewest phenolic compounds determined as compared to the other samples. Based on the chromatographic analyses, we were able to identify one anthocyanin and two phenolic acids (Figure 5 and Table 5). Phenolic acids were the most dominant class, where the primary compound was peak 3 assigned to 5-caffeoylquinic acid (chlorogenic acid) ([M − H]^+^, *m*/*z* = 355). The other phenolic acid at peak 1, with a molecular ion of [M − H]^+^, *m*/*z* = 139, was identified as hydroxybenzoic acid. Our data were in agreement and supported by several studies [36,37,38]. However, according to the literature, eggplant contains a significant number of phenolic acids, and besides those that were identified in this study, there were several derivatives of caffeoylquinic acid previously reported [35]. Additionally, other phenolic acids have also been identified: dicaffeoylquinic acid, feruoylquinic acid, and caffeoylshikimic. Besides the anthocyanin that was identified in this study, other delphinidin derivatives, such as delphinidin-3-rutinoside-5-glucoside, delphinidin-3-*O*-glucoside, and delphinidin-3-caffeoylrutinoside-5-glucoside were also reported in the literature [36,38].

The identified anthocyanin, at peak 2, was a glycosylated delphinidin represented by delphinidin-3-*O*-(6″-p-coumaroyl-glucoside). The representative phenolic compounds in eggplant are delphinidins, but especially the acylated one, as identified in this study. They are found in a high concentration in eggplant and they are used as a natural blue dye since they are more stable than other anthocyanins. In addition to anthocyanins being stable in acidic pH, acylated delphinidin derivatives are also stable in neutral pH levels, which make them better candidates for their use as dyes. This is a crucial benefit in food processing, where the pH that is required to achieve the desired color cannot be ensured all the times [39].

**Figure 6:**
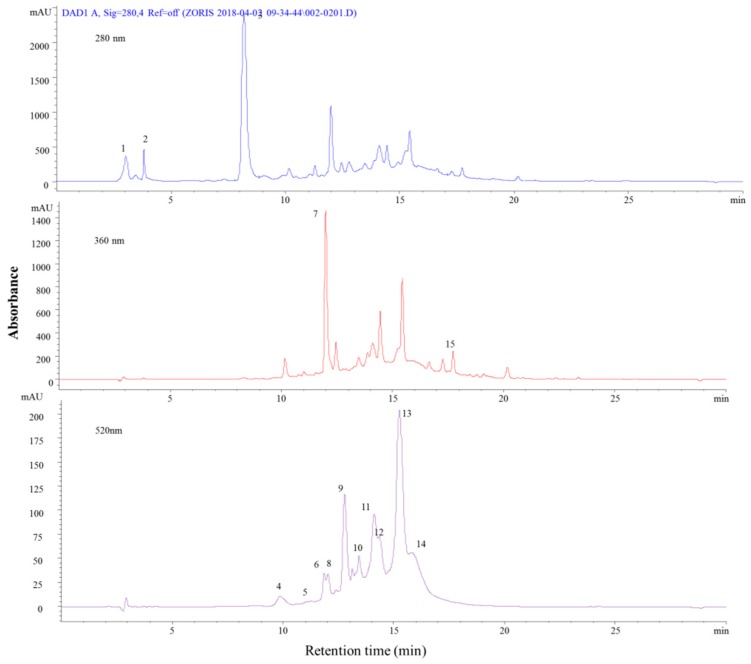
HPLC-PDA chromatograms of purple sweet potato recorded at 280, 360, and 520 nm.

Purple sweet potato was found to contain 15 phenolic compounds, where the most of them were anthocyanins (Figure 6 and Table 6). The identified anthocyanins were the derivatives of cyanidin and peonidin, accounting for a total of 10 anthocyanins. The complex structural compounds identified were: cyanidin-3-p-hydroxybenzoylsophoroside-5-glucoside ([M − H]^+^, *m*/*z* = 893) (peak 6), followed by peonidin-3-p-hydroxybenzoylsophoroside-5-glucoside ([M − H]^+^, *m*/*z* = 907 (peak 8), cyanidin-3-caffeoyl-p-hydroxybenzoylsophoroside-5-glucoside ([M − H]^+^, *m*/*z* = 1005) (peak 11), and peonidin-3-caffeoyl-p-hydroxybenzoylsophoroside-5-glucoside ([M − H]^+^, *m*/*z* = 1069) (peak 12). The next class of compounds was phenolic acids, where five compounds were identified. The most important and often common phenolic acids were: hydroxybenzoic acid ([M − H]^+^, *m*/*z* = 139), chlorogenic acid ([M − H]^+^, *m*/*z* = 355), and ferulic acid ([M − H]^+^, *m*/*z* = 195). Our results for the identity of the phenolic compounds that were found in purple sweet potato were in agreement available published data [40,41,42]. According to literature, the anthocyanins identified in purple sweet potato were only derivatives of cyanidins and peonidins, as confirmed in our study, but the authors [42] identified a few other derivatives, such as cyanidin-3-sophoroside-5-glucoside, cyanidin-3-(6″,6″-dicaffeoylsophoroside)-5-glucoside, peonidin-3-(6″caffeoyl-p-hidroxybenzoylsophoroside)-5-glucoside, cyanidin 3-caffeoyl-p-coumarylsophoroside-5-glucoside, and peonidin-3-feruloyl-p-coumarylsophroside-5-glucoside.

**Figure 7:**
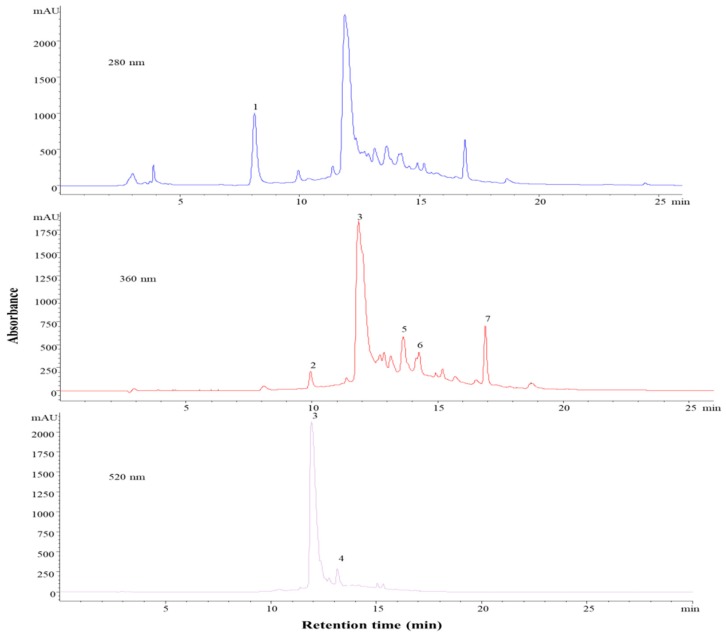
HPLC-PDA chromatograms of black carrot recorded at 280, 360 and 520 nm.

The black carrot was found to Figure 7 and Table 7. Most of the identified compounds were phenolic acids, of which the most common were: 5-caffeoylquinic acid (chlorogenic acid) ([M − H]^+^, *m*/*z* = 355), syringic acid (dimethoxy-4-hydroxybenzoic acid) ([M − H]^+^, *m*/*z* = 198), caffeic acid ([M − H]^+^, *m*/*z* = 181), and feruloylquinic acid ([M − H]^+^, *m*/*z* = 369).

The other phenolic acids present were 3-caffeoylquinic acid (neochlorogenic acid) ([M − H]^+^, *m*/*z* = 355), and 3,5-dicaffeoylquinic acid ([M − H]^+^, *m*/*z* = 517). The next class of investigated compounds was the anthocyanins, where the two major compounds were cyanidin-3-(p-coumaroyl)-diglucoside-5-glucoside ([M − H]^+^, *m*/*z* = 919 and cyanidin-3-(feruloyl)-glucoside-5-glucoside ([M − H]^+^, *m*/*z* = 787). The relevant studies considered [43,44,45,46] that our data are consistent with the literature, where no flavonoid was identified. Additionally, no other anthocyanins other than cyanidins were identified. Additional phenolic compounds were identified in black carrot in a previous study as cyanidin-3-xylosyl-glucosyl-galactoside, cyanidin-3-xylosyl- sinapoyl-glucosyl-galactoside, cyanidin-3-xylosyl-feruloyl-glucosyl-galactoside, and cyanidin-3-xylosyl-coumaroyl-glucoside-galactoside [43].

### 2.4. LC-MS Quantification Analysis 

The identified compounds were divided into phenolic acids, flavonoids, and anthocyanins. All samples contained a high amount of phenolics and antioxidant activity potential. The total amount of phenolic compounds in red chicory extract was 201.77 mg/100 g FW, where the flavonols were abundant (135.4 mg/100 g FW). Among the flavonols, the major component was quercetin 3,4-*O*-diglucoside (82.5 mg/100 g FW). Anthocyanins were in a relatively high amount (39.2 mg/100 g FW), where cyanidin-3-*O*-glucoside 30.9 mg/100 g FW). Among the most abundant phenolic acids, hydroxybenzoic acids (14.81 mg/100 g FW) and protocatechuic acid (6.6 mg/100 g FW) were in the highest amounts. Table 8 summarizes the level of the identified compounds. In a recent study, multiple varieties of red chicory were analyzed and the hydroxycinnamic acid concentration was found to be ranging between 47 mg and 362 mg/100 g FW, where chlorogenic acid was found to be the most substantial amount, ranging from 14.2 mg to 89.5 mg/100 g. The total amount of flavonols was between 1.7 mg to 19.9 mg/100 g FW, where it represented a minority class, as expected. Moreover, the same study reported anthocyanins only in two varieties of red chicory, where they were in minimal amounts of 0.2 mg to 2.7 mg /100 g FW [47].

In red onion, 56.5 mg/100 g FW of phenolic compounds were determined, where the phenolic acids were in the highest concentration (25.9 mg/100 g FW). Syringic acid was demonstrated to be a major compound, being the only phenolic acid that was identified and found in an amount of 25.9 mg/100 g FW. Quercetin derivatives was identified as another category of compounds with an approximate amount to phenolic acids (25.1 mg/100 g FW), with quercetin-3,4-*O*-diglucoside being the major compound (19.5 mg/100 g FW). Red onion was also found to contain anthocyanins at the level of 5.5 mg/100 g FW, where cyanidin-3-*O*-glucoside and cyanidin-3-*O*-laminaribioside (4.3 mg/100 g FW) were the major compounds.

Table 9 provides all of the concentration values of the identified compounds. In a study that was conducted on several varieties of onion, the primary compound reported was quercetin, where it was consistently high in all varieties, ranging from 7.7 to 46.3 mg /100 g FW. This can also be observed in Table 9, where flavonols and particularly quercetin were in the most considerable quantities [48].

Eggplant extracts had a concentration of 76.7 mg/100 g FW of phenolic compounds. Phenolic acids represented the most abundant class of compounds, where 5-caffeoylquinic acid (chlorogenic acid), with a concentration of 62.1 mg/100 g FW, was the major compound. Besides these compounds, an anthocyanin (delphinidin-3-*O*-(6″-p-coumaroyl-glucoside)) which is an important compound in the natural dye industry, was also present in a concentration of 8.7 mg/100 g FW. Table 10 shows all levels of the identified compounds. 

The total amount of phenolic compounds in purple sweet potato was 234.8 mg/100 g FW. Phenolic acids made up the highest portion under phenolic compounds, with the amount of 192.5 mg/100 g FW, where the major compound was syringic acid (97.0 mg/100 g FW). It is a part of the hydroxybenzoic acids class (119.3 mg/100 g FW), which are found in a higher amount than the class of hydroxycinnamic acids. Additionally, hydroxycinnamic acids (73.1 mg/100 g FW) were found in a high amount, where the major compound was chlorogenic acid (60.9 mg/100 g FW). 

Among anthocyanins (42.3 mg/100 g FW), the major compound was peonidin-3-caffeoyl-p-hydroxybenzoylsophoroside-5-glucoside (15.2 mg/100 g FW), and their concentration was lower when compared to the other phenolic compounds that were quantified in purple sweet potato. Table 11 provides all values of the identified phenolic compounds.

Black carrot contained a concentration of 56.8 mg/100 g FW phenolic compounds. Phenolic acids were found in the highest amount, of which hydroxycinnamic acids (34.3 mg/100 g FW) had the highest concentration. The major compound was (5-caffeoylquinic acid) chlorogenic acid, which was found in a quantity of 21.7 mg/100 g FW). Of the phenolic acids, caffeic acid was also abundant, 5.3 mg/100 g FW. The following class of compounds was anthocyanins, where cyanidin-3-(p-coumaroyl)-diglucoside-5-glucoside was found in a concentration of 21.7 mg/100 g FW. Table 12 presents all of the values of the identified compounds.

### 2.5. Statistical Analysis

**Figure 8:**
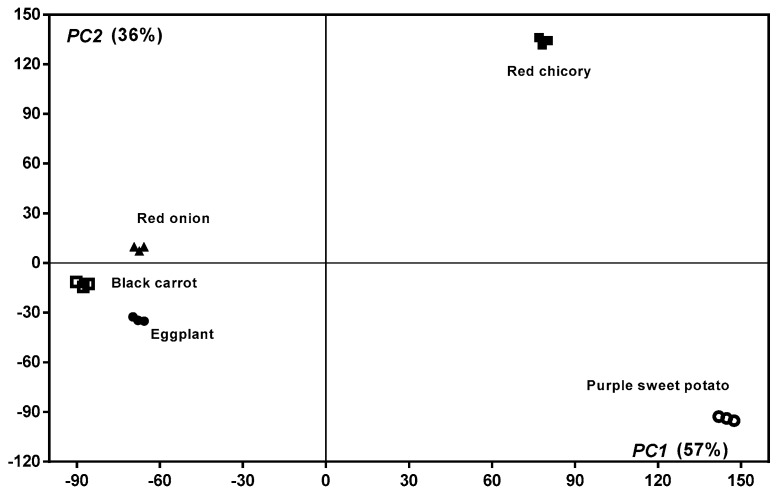
Principal component analysis of spectrophotometric and chromatographic data.

The data that were obtained from the spectrophotometrical analysis of the samples, as well as the ones from the chromatographic analysis (polyphenols profiles), were subject to the principal component analysis (PCA) (Figure 8). This chemometric method is a valuable tool in establishing interrelationships between different variables, allowing for the detection and interpretation of samples patterns, emphasizing their similarities and differences. Thus, the first two principal components explained 93% of the data variance, showing good discrimination between the studied samples. The variables with high relevance in sample differentiation include total phenolic content, total flavonols content, but also some specific phenols, such as hydroxybenzoic, dihydroxybenzoic, syringic, ferulic, and dihydroxy p-coumaric acids. Among anthocyanins, Cyanidin-3-*O*-glucoside and Cyanidin-3-*O*-(6″-malonyl-glucoside), together with Catechin, Quercetin-3,4-*O*-diglucoside, and Quercetin-3-*O*-glucoside can be considered to be marker compounds for the red chicory sample. 

The contributions of each type of polyphenols (phenolic acids, flavonoids and anthocyanins) to antioxidant activity determined by three various methods (CUPRAC, ABTS, DPPH) were calculated while using Pearson correlation coefficients (r). Table 13 provides the summary for correlations. Significant correlations were obtained between anthocyanins content and all of the antioxidant methods applied, especially for anthocyanins content and ABTS (r = 0.952) and DPPH (0.984). Flavonols were weakly correlated with high antioxidant potential on all three methods. No correlations were observed between both phenolic acids’ classes (HBA/HCA) and all antioxidant methods: HBA-CUPRAC (r = 0.113), HBA-ABTS (r = 0.390), HBA-DPPH (r = 0.515), also in case of HCA, the correlations were the following: HCA-CUPRAC (r = 0.204), HCA-ABTS (r = 0.356), and HCA-DPPH (r = 0.184). 

From these correlations, we can infer that anthocyanins were responsible for the high antioxidant activity of analyzed samples. The flavonols were found to be moderately responsible and both phenolic acids (HBA/HCA) were not responsible for antioxidant activity. 

## 3. Discussion 

The purpose of this study was to separate, identify, and quantify the phenolic compounds of red chicory, red onion, eggplant, purple sweet potato, and black carrot and evaluate the correlation between their phytochemical composition and antioxidant acuity. Antioxidant activity analysis was performed while using several methods (ABTS, CUPRAC, FRAP, and DPPH). Red chicory and purple sweet potato had the highest antioxidant activity due to the high content of phenolic compounds, especially flavonols (quercetin-3,4-*O*-diglucoside) in red chicory and anthocyanins (peonidin-3-caffeoyl p-hydroxybenzoylsophoroside-5 -glucoside) in purple sweet potato. Phenolic acids in all samples were the most common compounds found. Of the acid category, chlorogenic acid and syringic acid were more abundant. Among the anthocyanins, cyanidin was present in all of the samples. The rarest phenolic compounds found were: feruloylquinic acid (hydroxycinnamic acid) in black carrot and catechin (flavonol) in red chicory. We believe that this study has been able to identify many phenolic compounds that can be utilized in different industries, particularly in the food industry, where new information and resources are continually being sought. There is a need for healthy alternatives for coloring food and, among the compounds that were identified in this work, some can be evaluated as natural colorants.

## 4. Materials and Methods

### 4.1. Chemicals and Reagents 

All of the solvents and chemicals that were used in this study were purchased from Sigma-Aldrich (Darmstadt, Germany). 

The anthocyanin standards cyanidin-3-*O*-glucoside chloride, pelargonidin-3-*O*-glucoside chloride, cyanidin-3-*O*-galactoside (purity 90%), cyanidin-3-*O*-arabinoside (purity 97%), cyanidin-3-*O*-glucoside (purity 95%), and cyanidin (purity 95%) were purchased from Polyphenols AS (Sandnes, Norway). Chlorogenic acid, caffeic acid, quercetin-3-*O*-rutinoside, quercetin-3-*O*-glucoside, ellagic acid, rutin, and myricetin were also purchased from Sigma-Aldrich (Darmstadt, Germany).

### 4.2. Plant Material and Extract Preparation

Eggplant, red onion, black carrot, purple sweet potato, and red chicory were purchased from a local market in Cluj-Napoca, Romania. The vegetables were stored at −18 °C until further analysis. Acidified methanol (MeOH + 0.03% HCl) was prepared before proceeding the extraction, and the samples were fine grounded in a mortar. Subsequently, 10 grams of the grounded sample were mixed with the solvent. The colored mixture was centrifuged at 3214 g, the supernatant was collected, and the extraction procedure was repeated until the samples turned colorless. Further, the collected extracts were evaporated at 40 °C under reduced pressure (Rotavapor R-124, Buchi, Switzerland), dissolved in a known amount of acidified water, and filtered through 0.45 μm Millipore filter before all of the qualitative and quantitative analyses. The extractions were carried out at room temperature.

### 4.3. Determination of Total Phenolic Content

The total phenolic content (TPC) of all the extracts was determined following the Folin–Ciocalteu spectrophotometric method with small adaptations, as described by Singleton [49]. Briefly, the sample (10 μL) (standard/extract) was mixed with 1800 μL distilled water, Folin–Ciocalteu reagent (120 µL), and Na_2_CO_3_ (7.5% in water) (340 µL). After 60 minutes incubation in the dark and at room temperature, the absorbance of the samples was measured at 750 nm. The results were expressed as mg of gallic acid equivalents (GAE) per 100 g of fresh weight (FW).

### 4.4. Determination of Total Flavonoid Content

The total flavonoid content of the samples was determined according to the aluminum chloride colorimetric method, based on the formation of a complex between the flavonoid and aluminum described by Zhishen [50]. The extracts were mixed with 5% NaNO_2_ (90 µL), AlCl_3_ (90 µL), NaOH (600 µL), and 720 µL of distilled water. After the solution was mixed well, the absorbance was measured at 510 nm. Total flavonoid content was expressed as mg quercetin equivalents/100 g of fresh weight (FW). 

### 4.5. ABTS (ABTS Radical Cation Decolorization Assay) Radical Scavenging Assay

This assay is based on the capacity of an antioxidant to scavenge the ABTS radical cation (ABTS^+^) as compared to a standard antioxidant (Trolox). The protocol was adapted to 96 wells microplate, as described by Arnao [51]. The ABTS solution was prepared from a stock solution of 7mM ABTS and 2.45 mM potassium persulfate and kept at dark and room temperature for 12–16 h. ABTS was diluted with EtOH until the solution had an absorbance value of 0.700 ± 0.02 at 734 nm before its use in order to obtain the working solution from the stock solution. In a 96-well microplate, the samples and Trolox standard (20 µL) were combined with the working solution (170 µL). After 6 min of incubation at 30 °C, the absorbance of the samples was measured at 734 nm. A standard curve was prepared using different concentrations of Trolox and the results were expressed as µM Trolox/g sample.

### 4.6. Cupric Ion Reducing Antioxidant Capacity (CUPRAC) Assay

The cupric ion reducing the antioxidant capacity of the extracts was determined according to a previously described method by Apak [52]. A mixture comprising 500 μL of CuCl_2_ (10 mM), 500 μL neocuproine alcoholic solution (7.5 mM), and 500 μL ammonium acetate buffer (1M, pH 7.0), 0.49 mL water, and 10 μL extract was left to stand at room temperature for 30 min. The absorbance was recorded using a spectrophotometer (JASCO V-630 series, International Co., Ltd., Japan) at 450 nm against the blank. A standard curve was prepared using different concentrations of Trolox and the results were expressed as μmol Trolox/100 g FW. 

### 4.7. DPPH Assay Scavenging Activity Assay

DPPH scavenging assay was conducted according to the method that was reported by Brand–Williams [53]. The working solution was prepared fresh in ethanol. 250 μL of the DPPH solution was mixed with 35 μL of the sample and incubated for 30 minutes, and then the absorbance was measured at 515 nm. A standard curve was prepared using different concentrations of Trolox and the results were expressed as µmol Trolox/100 g FW.

### 4.8. HPLC-PDA/-ESI^+^-MS Identification and Quantification of Phenolic Compounds

ESI-MS (Electrospray ionization coupled with mass spectrometry) analysis was performed on an Agilent 1200 system (Chelmsford, MA, USA) that was equipped with a binary pump delivery system LC-20 AT (Prominence), a degasser DGU-20 A3 (Prominence), and a diode array SPD-M20 UV–VIS detector (DAD). The separation of the compounds was achieved on an Eclipse XDB C18 column (4 μm, 4.6 × 150 mm). The mobile phases consisted of solvent A—bidistilled water and 0.1% acetic acid/acetonitrile (99/1) v/v, while solvent B was acetonitrile and acetic acid 0.1%. The gradient elution system was programmed, as following: 0–2 min, isocratic with 5% (v/v) eluent B; 2–18 min, linear gradient from 5% to 40% (v/v) eluent B; 18–20 min, linear gradient from 40% to 90% (v/v) eluent B; 20–24 min, isocratic on 90% (v/v) eluent B; 24–25 min, linear gradient from 90% to 5% (v/v) eluent B; 25–30 min, isocratic on 5% (v/v) eluent B. Flow rate was set to 0.5 mL/min and column temperature was maintained at 25 °C. The chromatograms were monitored at 280, 360, and 520 nm. Identification of the compounds and peak assignments were done using their retention time, UV-VIS, and mass spectra, and also when comparing with the commercial standards (chlorogenic acid, caffeic acid, quercetin-rutinoside, quercetin-glucoside, ellagic acid, myricetin) and previously published literature. A single quadrupole 6110 mass spectrometer (Agilent Technologies, Chelmsford, MA, USA) equipped with an ESI probe was used for the mass spectrometric measurements. Measurements were performed in the positive mode with an ion spray voltage of 3000 V and a capillary temperature of 350 °C. Data were collected in full scan mode within the range 280 to 1000 *m*/*z*. For the quantification of anthocyanins, phenolic acids, and flavonoids, standard curves of cyanidin-3-*O*-galactoside, chlorogenic acid, and rutin were used, respectively, and expressed as mg/100 g FW. 

### 4.9. Statistical Analysis

The statistical analysis of the results (spectrophotometric and chromatographic data) was performed by principal component analysis (PCA) with cross-validation (full model size and center data). In order for all of the variables included in the analysis to have an equal chance to influence the model, the standardization was used as the scaling technique. All of the statistical analyses were performed using Unscrambler X Version 10.1 software (CAMO Software AS, Oslo, Norway).

Linear Pearson correlation coefficient was also used to analyze the strength of correlation between the results that were obtained for antioxidants assays. 

## 5. Conclusions

The present study reveals important information regarding the phytochemical composition of the most consumed reddish vegetables in our diet. The analyzed samples were found to be rich in polyphenols, such as anthocyanins, flavonols, HBA, and HCA. The Linear Pearson correlation coefficient was performed to evaluate the correlation between the quantitative analysis of each type of phenolic compounds and their antioxidants activities. The obtained results clearly showed a correlation between the high concentration of anthocyanins and high antioxidant activity.

Anthocyanins are vibrant natural pigments that are found in nature and their color is defined by the proportions of the different anthocyanin forms, namely the red flavylium cation, the violet quinonoidal bases, the colorless water or sulfite adducts, and the yellow chalcones. To date, anthocyanins have had various applications, not only in the technological field as natural colorants, but also in the field of human health, since they are one of the bioactive components used as nutraceuticals and in traditional medicine. As a nutraceutical, the bioavailability of anthocyanin is the key factor for maintaining good health and in the prevention of degenerative diseases. Anthocyanins are of particular interest to the food colorant industry due to their ability to impart vibrant colors to the product; therefore, anthocyanins from various natural sources are used as food colorants in foods and beverages. The application of anthocyanin-based colorants in fruit yogurt and many types of fruit-flavored dry mixes is becoming more popular. 

Besides the nutritional value and the health benefits due to the antioxidant potential, the five analyzed vegetables also have a high scientific value due to a large number of compounds and their variability, opening new opportunities in many other areas, such as the food industry, dye industry, pharmaceutical, and many others. 

## Figures and Tables

**Table 1:** Total Phenolics and Flavonoids content of the selected vegetables.

	Analysi	Total Phenolics mg GAE/100 g FW *	Total Flavonoids mg QUE/100 g FW **
Sample			
**Red chicory**		216.15 ± 2.4	95.48 ± 0.9
**Red onion**		141.14 ± 1.7	61.95 ± 0.6
**Eggplant**		134.23 ± 1.3	101.79 ± 3.4
**Purple sweet potato**		167.47 ± 1.04	72.24 ± 2.2
**Black carrot**		189.50 ± 1.5	22.14 ± 1.3

* Expressed as mg gallic acid equivalents/100 g FW; ** Expressed as mg rutin equivalents/100 g fresh weight (FW).

**Table 2:** Spearman’s rank correlation coefficient for antioxidant methods.

Methods			(CUPRAC)	ABTS	DPPH
Spearman’s rho	CUPRAC	Correlation Coefficient	1.000	0.914 **	0.918 **
		Sig. (2-tailed)	.	0.000	0.000
		N	15	15	15
	ABTS	Correlation Coefficient	0.914 **	1.000	0.946 **
		Sig. (2-tailed)	0.000	.	0.000
		N	15	15	15
	DPPH	Correlation Coefficient	0.918 **	0.946 **	1.000
		Sig. (2-tailed)	0.000	0.000	.
		N	15	15	15

**. Correlation is significant at the 0.01 level (two-tailed).

**Table 3:** Characterization of phenolic compounds in red chicory via positive mass spectrometry LC- Mass Spectrometry (MS).

Peak No.	R_t_ (min)	[M + H]^+^ (*m*/*z*)	UV λ_max_ (nm)	Tentatively Identified Compound
1	2.95	139	265	Hydroxybenzoic acid
2	3.79	156	260	Dihydroxybenzoic acid
3	6.26	167	270	Dihydroxy p-coumaric acid
4	8.03	155	290	Protocatechuic acid
5	11.10	449, 207	280, 517	Cyanidin-3-*O*-glucoside
6	11.90	355	320	Chlorogenic acid
7	12.23	291	279	Catechin
8	12.93	535, 287	280, 516	Cyanidin-3-*O*-(6″-malonyl-glucoside)
9	15.45	611, 303	250, 350	Quercetin-3-*O*-rutinoside (Rutin)
10	15.71	625,463,303	250, 350	Quercetin-3,4-*O*-diglucoside
11	16.15	465, 303	250, 360	Quercetin-3-*O*-glucoside
12	16.57	551, 303	255, 360	Quercetin-3-*O*-(6″-malonyl-glucoside)

**Table 4:** Characterization of phenolic compounds in red onion via positive LC-MS.

Peak No.	R_t_ (min)	[M+H]^+^ (*m*/*z*)	UV λ_max_ (nm)	Tentatively Identified Compound
1	8.23	198	290	Syringic acid (Dimethoxy-4-hydroxybenzoic acid)
2	11.23	449, 611	280, 519	Cyanidin-3-*O*-glucoside & Cyanidin-3-*O*-laminaribioside
3	12.91	535, 697	280, 519	Cyanidin-3-(6″-malonyl-glucoside) & Cyanidin-3-(6″-malonyl-laminaribioside)
4	14.03	627, 465, 303	250, 360	Quercetin-3,4-*O*-diglucoside
5	17.65	465, 303	250, 360	Quercetin-3-*O*-glucoside

**Table 5:** Characterization of phenolic compounds in eggplant via positive LC-MS.

Peak No.	R_t_ (min)	[M+H]^+^ (*m*/*z*)	UV λ_max_ (nm)	Tentatively Identified Compound
1	2.95	139	265	Hydroxybenzoic acid
2	10.26	611, 303	280, 524	Delphinidin-3-*O*-rutinoside
3	11.89	355	320	5-Caffeoylquinic acid (Chlorogenic acid)

**Table 6:** Characterization of phenolic compounds in purple sweet potato via positive LC-MS.

Peak No.	R_t_ (min)	[M+H]^+^ (*m*/*z*)	UV λ_max_ (nm)	Tentative Identified Compound
1	2.95	139	265	Hydroxybenzoic acid
2	3.86	156	260	Dihydroxybenzoic acid
3	8.16	198	290	Dimethoxy-4-hydroxybenzoic acid (Syringic acid)
4	10.15	787	278, 516	Peonidin-3-*O*-sophoroside-5-*O*-glucoside
5	11.28	463	276, 520	Peonidin-3-*O*-glucoside
6	11.98	893	320, 512	Cyanidin-3-p-hydroxybenzoylsophoroside-5-glucoside
7	11.98	355	320	Chlorogenic acid
8	12.78	907	276, 518	Peonidin-3-p-hydroxybenzoylsophoroside-5-glucoside
9	13.47	935	287, 521	Cyanidin-3-caffeoylsophoroside-5-glucoside
10	14.11	949	281, 521	Peonidin-3-caffeoylsophoroside-5-glucoside
11	14.44	1055	280, 522	Cyanidin-3-caffeoyl-p-hydroxybenzoylsophoroside-5-glucoside
12	15.28	1111	281, 522	Peonidin-3-dicaffeoylsophoroside-5-glucoside
13	15.44	1069	324, 520	Peonidin-3-caffeoyl-p-hydroxybenzoylsophoroside-5-glucoside
14	15.81	1125	301, 519	Peonidin-3-caffeoy-feruloylsophoroside-5-glucoside
15	16.75	195	328	Ferulic acid

**Table 7:** Characterization of phenolic compounds in black carrot via positive LC-MS.

Peak No.	R_t_ (min)	[M + H]^+^ (*m*/*z*)	UV λ_max_ (nm)	Compound
1	8.11	198	290	Syringic acid (Dimethoxy-4-hydroxybenzoic acid)
2	9.95	355	320	3-Caffeoylquinic acid (Neochlorogenic acid)
3	11.89	355, 919	320, 520 280, 520	5-Caffeoylquinic acid (Chlorogenic acid) Cyanidin-3-(p-coumaroyl)-diglucoside-5-glucoside
4	13.14	787	287, 520	Cyanidin-3-(feruloyl)-glucoside-5-glucoside
5	13.64	181, 163	320	Caffeic acid
6	14.25	369, 195	330	Feruloylquinic acid
7	16.91	517	328	Ferulic acid

**Table 8:** Total polyphenolic compounds content in red chicory.

Red Chicory		
Class of Compounds	Compound	mg/100 g FW
Anthocyanins	Cyanidin-3-*O*-glucoside Cyanidin-3-*O*-(6″-malonyl-glucoside)	30.91 8.289
**Total Anthocyanins**		**39.20 ^1^**
Flavonols	Catechin Quercetin-3-*O*-rutinoside (Rutin) Quercetin-3,4-*O*-diglucoside Quercetin-3-*O*-glucoside Quercetin-3-*O*-(6″-malonyl-glucoside)	4.71 5.69 82.55 32.59 9.86
**Total Flavonols**		**135.41 ^2^**
Hydroxybenzoic acid (HBA)	Protocatechuic acid Hydroxybenzoic acid Dihydroxybenzoic acid	6.63 5.75 2.42
**Total HBA**		**14.81 ^3^**
Hydroxycinnamic acid (HCA)	Dihydroxy p-coumaric acid Chlorogenic acid	7.65 4.71
**Total HCA**		**12.36 ^3^**
**Total phenolic compound**		**201.77**

^1^ expressed as mg cyanidin-3-*O*-galactoside/100 g FW; ^2^ expressed as mg rutin/100 g FW; ^3^ expressed as mg chlorogenic acid /100 g FW.

**Table 9:** Total polyphenolic compounds content in red onion.

Red Onion		
Class of Compounds	Compound	mg/100 g FW
Anthocyanins	Cyanidin-3-*O*-glucoside+ Cyanidin-3-*O*-laminaribioside Cyanidin-3-(6″-malonyl-glucoside)+ Cyanidin-3-(6″-malonyl- laminaribioside)	4.38 1.18
**Total Anthocyanins**		**5.56 ^1^**
Flavonol	Quercetin-3,4-*O*-diglucoside Quercetin-3-*O*-glucoside	19.51 5.59
**Total Flavonol**		**25.11 ^2^**
Hydroxybenzoic acid (HBA)	Syringic acid	25.90
**Total HBA**		**25.90 ^3^**
**Total phenolic compound**		**56.58**

^1^ expressed as mg cyanidin/100 g FW; ^2^ expressed as mg rutin/100 g FW; ^3^ expressed as gallic acid/100 g FW.

**Table 10:** Total polyphenolic compounds content in eggplant.

Eggplant		
Class of Compounds	Compound	mg/100 g FW
Anthocyanins	Delphinidin-3-*O*-(6″-p-coumaroyl-glucoside)	8.72
**Total Anthocyanins**		**8.72 ^1^**
Hydroxybenzoic acid (HBA)	Hydroxybenzoic acid	5.89
**Total HBA**		**5.89 ^3^**
Hydroxycinnamic acid (HCA)	5-Caffeoylquinic acid (Chlorogenic acid)	62.15
**Total HCA**		**62.15**
**Total phenolic compound**		**76.77 ^3^**

^1^ expressed as mg cyanidin-3-*O*-galactoside/100 g FW; ^3^ expressed as mg chlorogenic acid /100 g FW.

**Table 11:** Total polyphenolic compounds content in purple sweet potato.

Purple Sweet Potato		
Class of Compounds	Compound	mg/100 g FW
Anthocyanins	Peonidin-3-*O*-sophoroside-5-*O*-glucoside Peonidin-3-*O*-glucoside Cyanidin-3-p-hydroxybenzoylsophoroside-5-glucoside Peonidin-3-p-hydroxybenzoylsophoroside-5-glucoside Cyanidin-3-caffeoylsophoroside-5-glucoside Peonidin-3-caffeoylsophoroside-5-glucoside Cyanidin-3-caffeoyl-p-hydroxybenzoylsophoroside-5-glucoside Peonidin-3-dicaffeoylsophoroside-5-glucoside Peonidin-3-caffeoyl-p-hydroxybenzoylsophoroside-5-glucoside Peonidin-3-caffeoy-feruloylsophoroside-5-glucoside	0.75 0.48 0.99 3.40 0.43 3.94 2.43 10.20 15.24 4.48
**Total Anthocyanins**		**42.37 ^1^**
Hydroxybenzoic acids (HBA)	Hydroxybenzoic acid Dihydroxybenzoic acid Dimethoxy-4-hydroxybenzoic acid (Syringic acid)	13.34 9.02 97.02
**Total HBA**		**119.39 ^3^**
Hydroxycinnamic acids (HCA)	Chlorogenic acid Ferulic acid	60.92 12.19
**Total HCA**		**73.11 ^3^**
**Total phenolic compound**		**234.88**

^1^ expressed as mg cyanidin-3-*O*-galactoside/100 g FW; ^3^ expressed as mg chlorogenic acid /100 g FW.

**Table 12:** Total polyphenolic compounds content in black carrot.

Black Carrot		
Class of Compounds	Compound	mg/100 g FW
Anthocyanins	Cyanidin-3-(p-coumaroyl)-diglucoside-5-glucoside Cyanidin-3-(p-coumaroyl)-diglucoside-5-glucoside	21.72 0.72
**Total Anthocyanins**		**22.45 ^1^**
Hydroxybenzoic acids (HBA)	Syringic acid (Dimethoxy-4-hydroxybenzoic acid)	0.035
**Total HBA**		**0.035 ^3^**
Hydroxycinnamic acids (HCA)	(5-Caffeoylquinic acid) Chlorogenic acid Feruloylquinic acid 3-Caffeoylquinic acid (Neochlorogenic acid) Caffeic acid Ferulic acid	21.73 3.043 1.283 5.346 2.937
**Total HCA**		**34.33 ^3^**
**Total phenolic compounds**		**56.82**

^1^ expressed as mg cyanidin-3-*O*-galactoside/100 g FW; ^3^ expressed as mg chlorogenic acid /100 g FW.

**Table 13:** Correlations between antioxidant activities and phenolic compounds (Anthocyanins. Flavonols, HBA, HCA).

Phenolic Compounds	Assays		
	CUPRAC	ABTS	DPPH
Anthocyanins	0.819	0.952	0.984
Flavonols	0.646	0.459	0.556
HBA	0.113	0.390	0.515
HCA	0.204	0.356	0.184

Pearson correlation coefficients at *p* < 0.05.

## Supplementary Materials

The following are available online.

## References

[1] Cheynier V., Tomas-Barberan F.A., Yoshida K. (2015). Polyphenols: From plants to a variety of food and nonfood uses. J. Agric. Food Chem..

[2] Mata R. (2007). Flavonoids, chemistry, biochemistry and applications by ø. M. Andersen (university of bergen) and k. R. Markham (industrial research ltd.). Crc press/taylor & francis, boca raton. 2006. Xiv + 1237 pp. 7 × 101/4 in. $249.95. Isbn 0-8493-2021-6. J. Nat. Prod..

[3] George B., Liaaen-Jensen S., Pfander H. (2009). Carotenoids Volume 5: Nutrition and Health.

[4] Wu S.B., Meyer R.S., Whitaker B.D., Litt A., Kennelly E.J. (2013). A new liquid chromatography-mass spectrometry-based strategy to integrate chemistry, morphology, and evolution of eggplant (solanum) species. J. Chromatogr. A.

[5] Goszcz K., Deakin S.J., Duthie G.G., Stewart D., Leslie S.J., Megson I.L. (2015). Antioxidants in cardiovascular therapy: Panacea or false hope?. Front. Cardiovasc. Med..

[6] Lisanti A., Formica V., Ianni F., Albertini B., Marinozzi M., Sardella R., Natalini B. (2016). Antioxidant activity of phenolic extracts from different cultivars of italian onion (allium cepa) and relative human immune cell proliferative induction. Pharm. Biol..

[7] Belwal T., Nabavi S.F., Nabavi S.M., Habtemariam S. (2017). Dietary anthocyanins and insulin resistance: When food becomes a medicine. Nutrients.

[8] Yang L., Ling W., Yang Y., Chen Y., Tian Z., Du Z., Chen J., Xie Y., Liu Z., Yang L. (2017). Role of purified anthocyanins in improving cardiometabolic risk factors in chinese men and women with prediabetes or early untreated diabetes-a randomized controlled trial. Nutrients.

[9] Mittal M., Siddiqui M.R., Tran K., Reddy S.P., Malik A.B. (2014). Reactive oxygen species in inflammation and tissue injury. Antioxid. Redox Signal..

[10] Hussain T., Tan B., Yin Y., Blachier F., Tossou M.C.B., Rahu N. (2016). Oxidative stress and inflammation: What polyphenols can do for us?. Oxid. Med. Cell. Longev..

[11] Zamora-Ros R., Knaze V., Rothwell J.A., Hémon B., Moskal A., Overvad K., Tjønneland A., Kyrø C., Fagherazzi G., Boutron-Ruault M.-C. (2016). Dietary polyphenol intake in europe: The european prospective investigation into cancer and nutrition (epic) study. Eur. J. Nutr..

[12] van Duynhoven J., Vaughan E.E., Jacobs D.M., A. Kemperman R., van Velzen E.J.J., Gross G., Roger L.C., Possemiers S., Smilde A.K., Doré J. (2011). Metabolic fate of polyphenols in the human superorganism. Proc. Natl. Acad. Sci. USA.

[13] Nicholson J.K., Holmes E., Kinross J., Burcelin R., Gibson G., Jia W., Pettersson S. (2012). Host-gut microbiota metabolic interactions. Science.

[14] Hall R.D., Brouwer I.D., Fitzgerald M.A. (2008). Plant metabolomics and its potential application for human nutrition. Physiol. Plant..

[15] Abbas Z.K., Saggu S., Sakeran M.I., Zidan N., Rehman H., Ansari A.A. (2015). Phytochemical, antioxidant and mineral composition of hydroalcoholic extract of chicory (cichorium intybus l.) leaves. Saudi J. Biol. Sci..

[16] Akanitapichat P., Phraibung K., Nuchklang K., Prompitakkul S. (2010). Antioxidant and hepatoprotective activities of five eggplant varieties. Food Chem. Toxicol..

[17] Han K.H., Sekikawa M., Shimada K., Hashimoto M., Hashimoto N., Noda T., Tanaka H., Fukushima M. (2006). Anthocyanin-rich purple potato flake extract has antioxidant capacity and improves antioxidant potential in rats. Br. J. Nutr..

[18] Toktas B., Bildik F., Ozcelik B. (2018). Effect of fermentation on anthocyanin stability and in vitro bioaccessibility during shalgam (salgam) beverage production. J. Sci. Food Agric..

[19] Zhang S.-L., Deng P., Xu Y.-C., LÜ S.-W., Wang J.-J. (2016). Quantification and analysis of anthocyanin and flavonoids compositions, and antioxidant activities in onions with three different colors. J. Integr. Agric..

[20] Shahidi F., Ambigaipalan P. (2015). Phenolics and polyphenolics in foods, beverages and spices: Antioxidant activity and health effects—A review. J. Funct. Foods.

[21] Zhang H., Tsao R. (2016). Dietary polyphenols, oxidative stress and antioxidant and anti-inflammatory effects. Curr. Opin. Food Sci..

[22] Prior R.L., Wu X., Schaich K. (2005). Standardized methods for the determination of antioxidant capacity and phenolics in foods and dietary supplements. J. Agric. Food Chem..

[23] Garcia E.J., Oldoni T.L., Alencar S.M., Reis A., Loguercio A.D., Grande R.H. (2012). Antioxidant activity by dpph assay of potential solutions to be applied on bleached teeth. Braz. Dent. J..

[24] Re R., Pellegrini N., Proteggente A., Pannala A., Yang M., Rice-Evans C. (1999). Antioxidant activity applying an improved abts radical cation decolorization assay. Free Radic. Biol. Med..

[25] Özyürek M., Güçlü K., Tütem E., Başkan K.S., Erçağ E., Esin Çelik S., Baki S., Yıldız L., Karaman Ş., Apak R. (2011). A comprehensive review of cuprac methodology. Anal. Methods.

[26] D’evoli L., Morroni F., Lombardi-Boccia G., Lucarini M., Hrelia P., Cantelli-Forti G., Tarozzi A. (2013). Red chicory (cichorium intybus l. Cultivar) as a potential source of antioxidant anthocyanins for intestinal health. Oxidative Med. Cell. Longev..

[27] Zhu F., Cai Y.Z., Yang X., Ke J., Corke H. (2010). Anthocyanins, hydroxycinnamic acid derivatives, and antioxidant activity in roots of different chinese purple-fleshed sweetpotato genotypes. J. Agric. Food Chem..

[28] Carazzone C., Mascherpa D., Gazzani G., Papetti A. (2013). Identification of phenolic constituents in red chicory salads (cichorium intybus) by high-performance liquid chromatography with diode array detection and electrospray ionisation tandem mass spectrometry. Food Chem..

[29] Sahan Y., Gurbuz O., Guldas M., Degirmencioglu N., Begenirbas A. (2017). Phenolics, antioxidant capacity and bioaccessibility of chicory varieties (cichorium spp.) grown in turkey. Food Chem..

[30] Petersson E.V., Liu J., Sjoberg P.J., Danielsson R., Turner C. (2010). Pressurized hot water extraction of anthocyanins from red onion: A study on extraction and degradation rates. Anal. Chim. Acta.

[31] D’Evoli L., Lucarini M., Potenza A., Ritota M., Sequi P., Lombardi-Boccia G. (2012). Anthocyanin profile of two italian cichorium intybus l. Cultivars. Acta Hortic..

[32] Innocenti M., Gallori S., Giaccherini C., Ieri F., Vincieri F.F., Mulinacci N. (2005). Evaluation of the Phenolic Content in the Aerial Parts of Different Varieties of *Cichorium Intybus* L.. J. Agric. Food Chem..

[33] Gennaro L., Leonardi C., Esposito F., Salucci M., Maiani G., Quaglia G., Fogliano V. (2002). Flavonoid and carbohydrate contents in tropea red onions: Effects of homelike peeling and storage. J. Agric. Food Chem..

[34] Tedesco I., Carbone V., Spagnuolo C., Minasi P., Russo G.L. (2015). Identification and quantification of flavonoids from two southern italian cultivars of allium cepa l., tropea (red onion) and montoro (copper onion), and their capacity to protect human erythrocytes from oxidative stress. J. Agric. Food Chem..

[35] García-Salas P., Gómez-Caravaca A.M., Morales-Soto A., Segura-Carretero A., Fernández-Gutiérrez A. (2014). Identification and quantification of phenolic compounds in diverse cultivars of eggplant grown in different seasons by high-performance liquid chromatography coupled to diode array detector and electrospray-quadrupole-time of flight-mass spectrometry. Food Res. Int..

[36] Niño-Medina G., Urías-Orona V., Muy-Rangel M.D., Heredia J.B. (2017). Structure and content of phenolics in eggplant (solanum melongena)—A review. S. Afr. J. Bot..

[37] Sadilova E., Stintzing F.C., Carle R. (2006). Anthocyanins, colour and antioxidant properties of eggplant (solanum melongena l.) and violet pepper (capsicum annuum l.) peel extracts. Z. Nat. C.

[38] Azuma K., Ohyama A., Ippoushi K., Ichiyanagi T., Takeuchi A., Saito T., Fukuoka H. (2008). Structures and antioxidant activity of anthocyanins in many accessions of eggplant and its related species. J. Agric. Food Chem..

[39] Ferrara L., Naviglio D. (2014). Nasunin, an antioxidant anthocyanin from eggplant peels, as natural dye to avoid food allergies and intolerances. Eur. Sci. J..

[40] Lee M.J., Park J.S., Choi D.S., Jung M.Y. (2013). Characterization and quantitation of anthocyanins in purple-fleshed sweet potatoes cultivated in korea by hplc-dad and hplc-esi-qtof-ms/ms. J. Agric. Food Chem..

[41] Tian Q., Konczak I., Schwartz S.J. (2005). Probing anthocyanin profiles in purple sweet potato cell line (ipomoea batatas l. Cv. Ayamurasaki) by high-performance liquid chromatography and electrospray ionization tandem mass spectrometry. J. Agric. Food Chem..

[42] Truong V.D., Deighton N., Thompson R.T., McFeeters R.F., Dean L.O., Pecota K.V., Yencho G.C. (2010). Characterization of anthocyanins and anthocyanidins in purple-fleshed sweetpotatoes by hplc-dad/esi-ms/ms. J. Agric. Food Chem..

[43] Garcia-Herrera P., Pérez-Rodríguez M.-L., Aguilera-Delgado T., Labari-Reyes M.-J., Olmedilla-Alonso B., Camara M., Pascual-Teresa S. (2016). Anthocyanin profile of red fruits and black carrot juices, purees and concentrates by hplc-dad-esi/ms-qtof. Int. J. Food Sci. Technol..

[44] Kamiloglu S., Ozkan G., Isik H., Horoz O., Van Camp J., Capanoglu E. (2017). Black carrot pomace as a source of polyphenols for enhancing the nutritional value of cake: An in vitro digestion study with a standardized static model. LWT.

[45] Kammerer D., Carle R., Schieber A. (2004). Quantification of anthocyanins in black carrot extracts (daucus carota ssp. Sativus var. Atrorubens alef.) and evaluation of their color properties. Eur. Food Res. Technol..

[46] Montilla E.C., Arzaba M.R., Hillebrand S., Winterhalter P. (2011). Anthocyanin composition of black carrot (daucus carota ssp. Sativus var. Atrorubens alef.) cultivars antonina, beta sweet, deep purple, and purple haze. J. Agric. Food Chem..

[47] Sinkovič L., Demšar L., Žnidarčič D., Vidrih R., Hribar J., Treutter D. (2015). Phenolic profiles in leaves of chicory cultivars (cichorium intybus l.) as influenced by organic and mineral fertilizers. Food Chem..

[48] Sellappan S., Akoh C.C. (2002). Flavonoids and antioxidant capacity of georgia-grown vidalia onions. J. Agric. Food Chem..

[49] Singleton V.L., Orthofer R., Lamuela-Raventós R.M. (1999). [14] analysis of total phenols and other oxidation substrates and antioxidants by means of folin-ciocalteu reagent. Methods in Enzymology.

[50] Zhishen J., Mengcheng T., Jianming W. (1999). The determination of flavonoid contents in mulberry and their scavenging effects on superoxide radicals. Food Chem..

[51] Arnao M.B., Cano A., Alcolea J.F., Acosta M. (2001). Estimation of free radical-quenching activity of leaf pigment extracts. Phytochem. Anal..

[52] Apak R., Güçlü K., Özyürek M., Esin Karademir S., Erçağ E. (2006). The cupric ion reducing antioxidant capacity and polyphenolic content of some herbal teas. Int. J. Food Sci. Nutr..

[53] Brand-Williams W., Cuvelier M.E., Berset C. (1995). Use of a free radical method to evaluate antioxidant activity. LWT-Food Sci. Technol..

