# Supplementary material for: Phytochemical Characterization of Five Edible Purple-Reddish Vegetables: Anthocyanins, Flavonoids, and Phenolic Acid Derivatives
Source: Molecules. 2019 Apr 18;24(8):1536. doi: 10.3390/molecules24081536 (PMC6514853; doi:10.3390/molecules24081536)
Supplement: Supplementary file 1 [file molecules-24-01536-s001.pdf]

Supporting Information

Article

Phytochemical Characterization of Five Edible Purple-Reddish Vegetables: Anthocyanins, Flavonoids, and Phenolic Acid Derivatives

Alexandra D. Frond [1,†], Cristian I. Iuhas [2,†], Ioana Stirbu [1], Loredana Leopold [1], Sonia Socaci [1], Stănilă Andreea [1], Huseyin Ayvaz [3], Socaciu Andreea [2], Socaciu Mihai [2], Socaciu Carmen [1] and Zorița Diaconeasa [1,*]

[1] Faculty of Food Science and Technology, University of Agricultural Sciences and Veterinary Medicine Cluj-Napoca, 3-5 Calea Manastur, Cluj-Napoca 400372, Romania, frondalexandradenisa@gmail.com (A.D.F.); ioanaalexandrastirbu@gmail.com (I.S.); loredana.leopold@usamvcluj.ro (L.L.); sonia.socaci@usamvcluj.ro (S.S.); andreea.stanila@usamvcluj.ro (S.A.); carmen.socaciu@usamvcluj.ro (S.C.)

[2] Faculty of Medicine, Iuliu Hatieganu University of Medicine and Pharmacy, Cluj-Napoca 400372, Romania, iuhascristianioan@yahoo.co.uk (C.I.I.); medisyn@yahoo.com (S.A.), socacium@yahoo.com (S.M.)

[3] Faculty of Engineering, Canakkale Onsekiz Mart University, Canakkale 17020, Turkey, huseyinayvaz@comu.edu.tr

* Correspondence: zorita.sconta@usamvcluj.ro; Tel.: +40-751-033-871

† These authors contributed equally to this work.

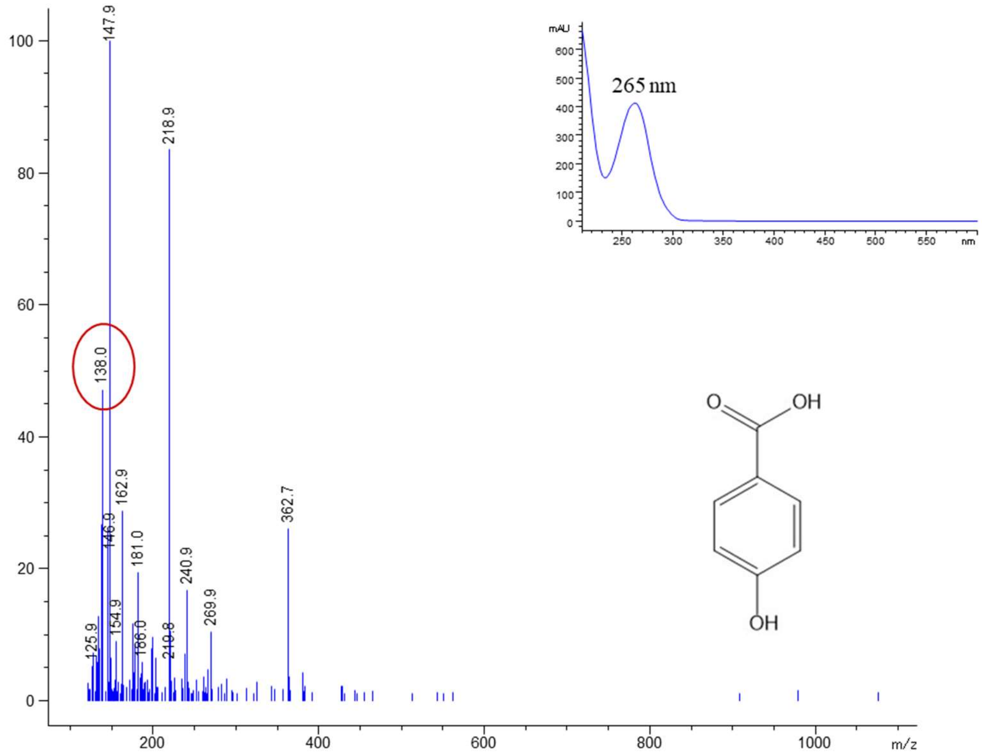

(a) peak 1
Hydroxybenzoic acid

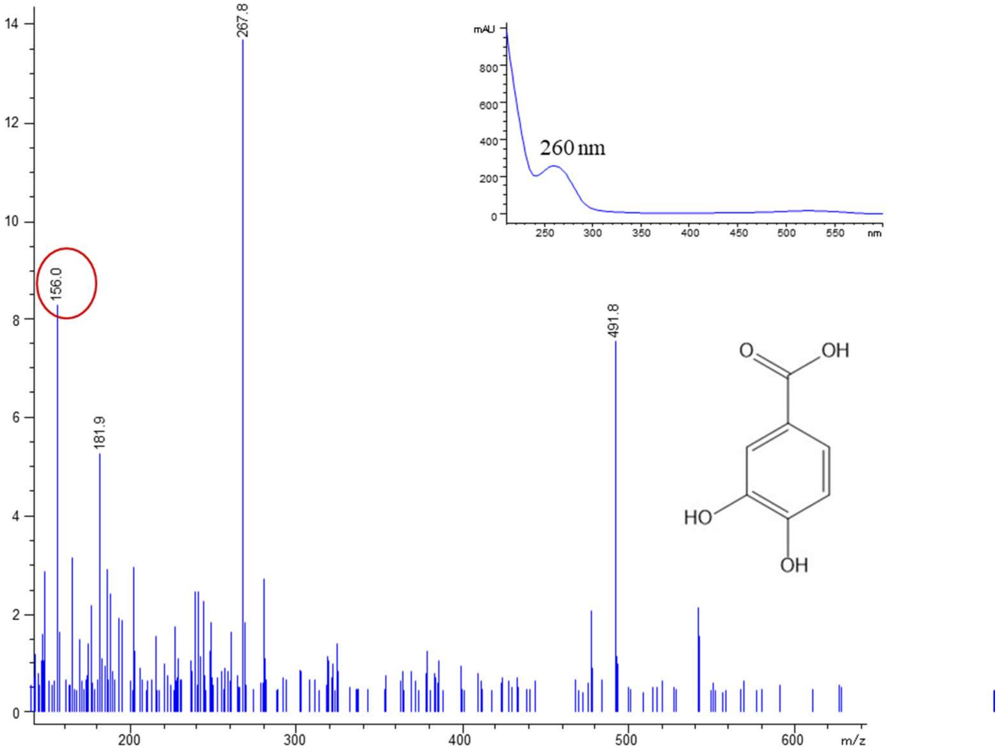

(b) peak 2
Dihydroxybenzoic
acid

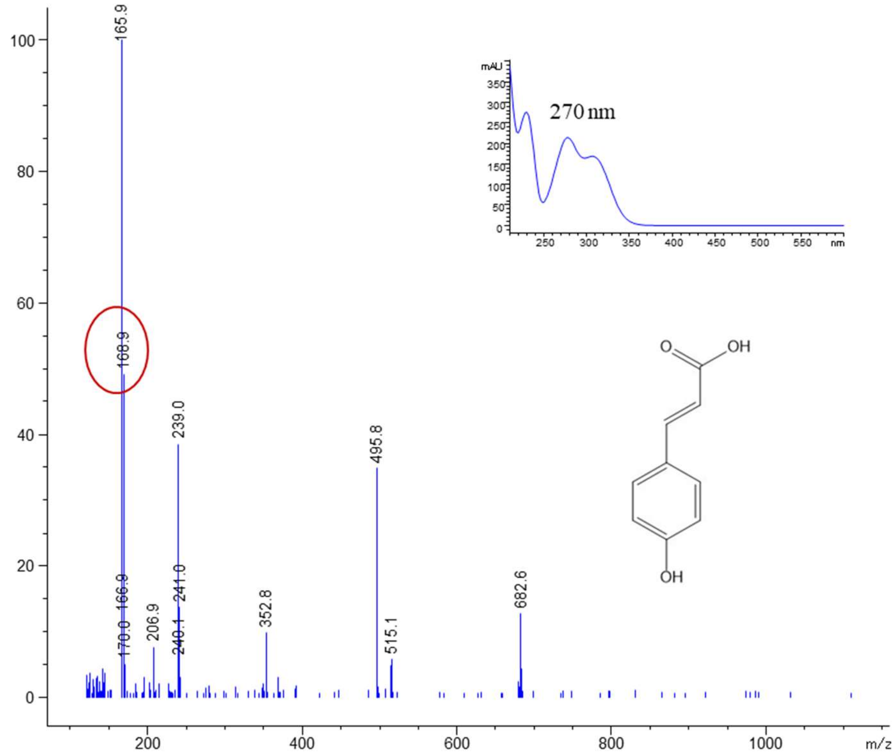

(c) peak 3
Dihydroxy p-coumaric acid

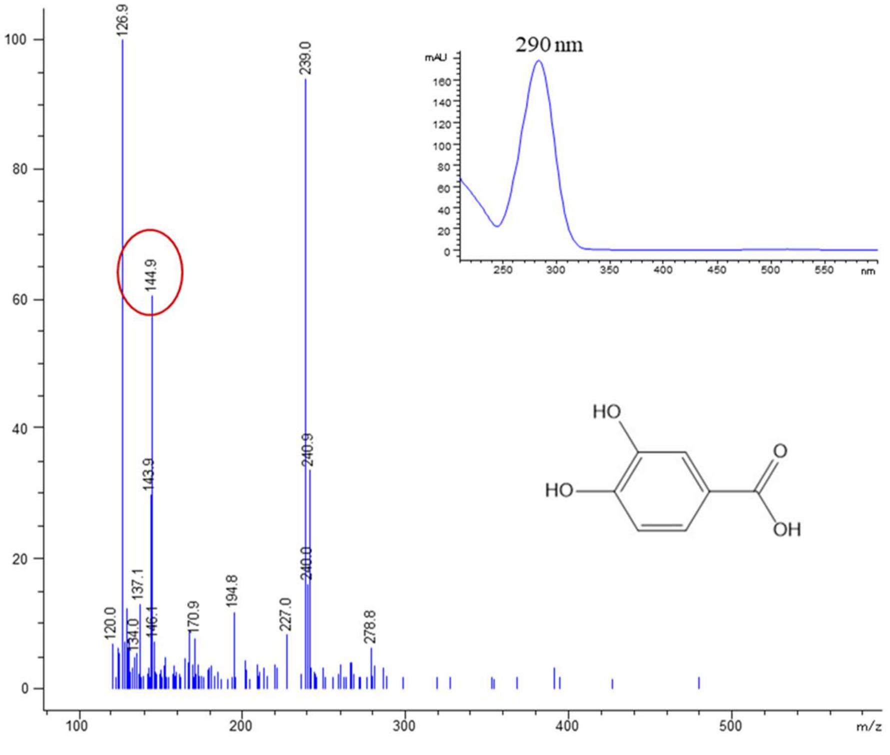

(d) peak 4
Protocatechuic acid

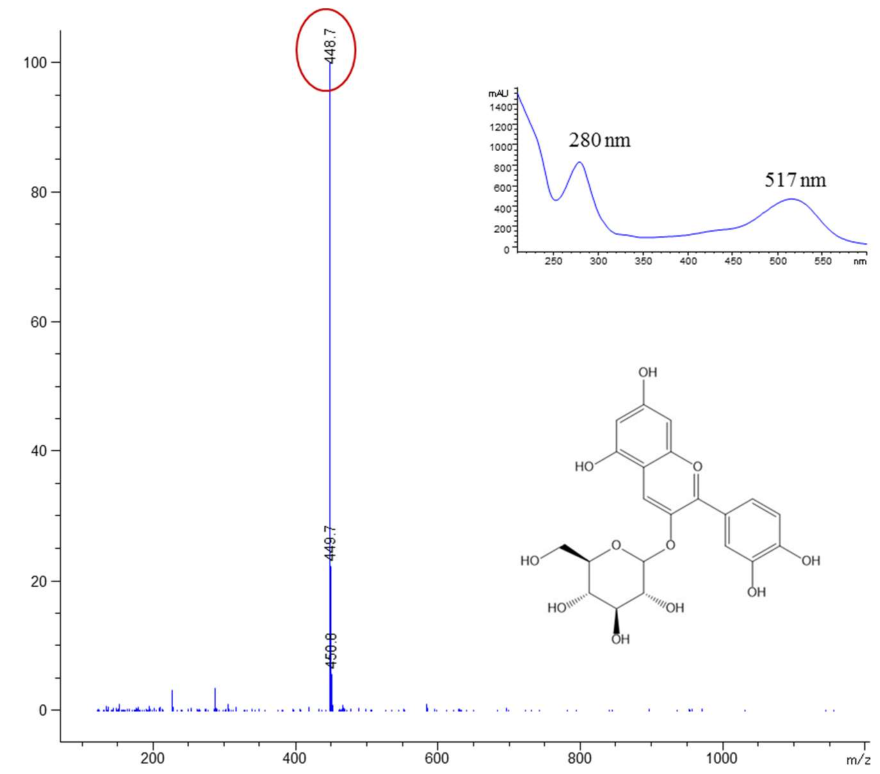

(e) peak 5
Cyanidin-3-O-glucoside

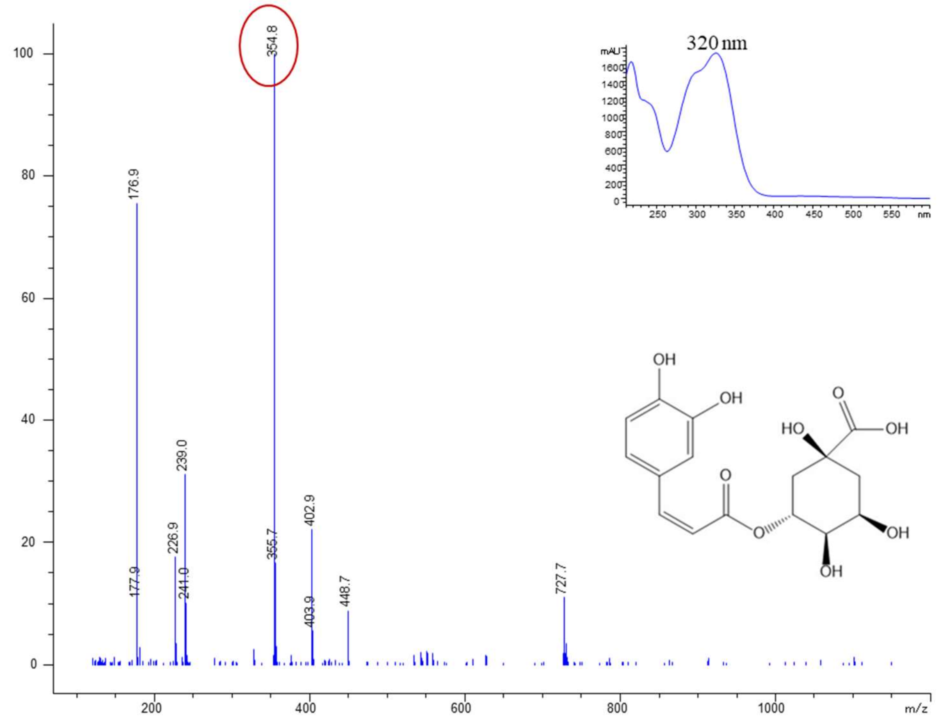

(f) peak 6
Chlorogenic acid

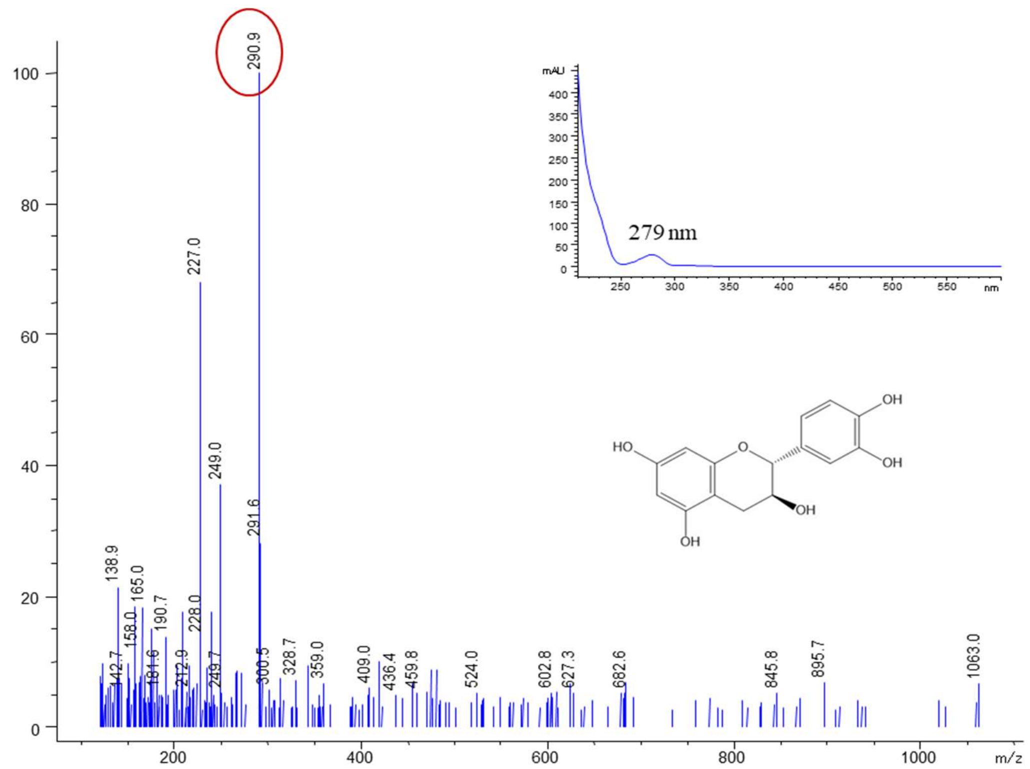

(g) peak 7
Catechin

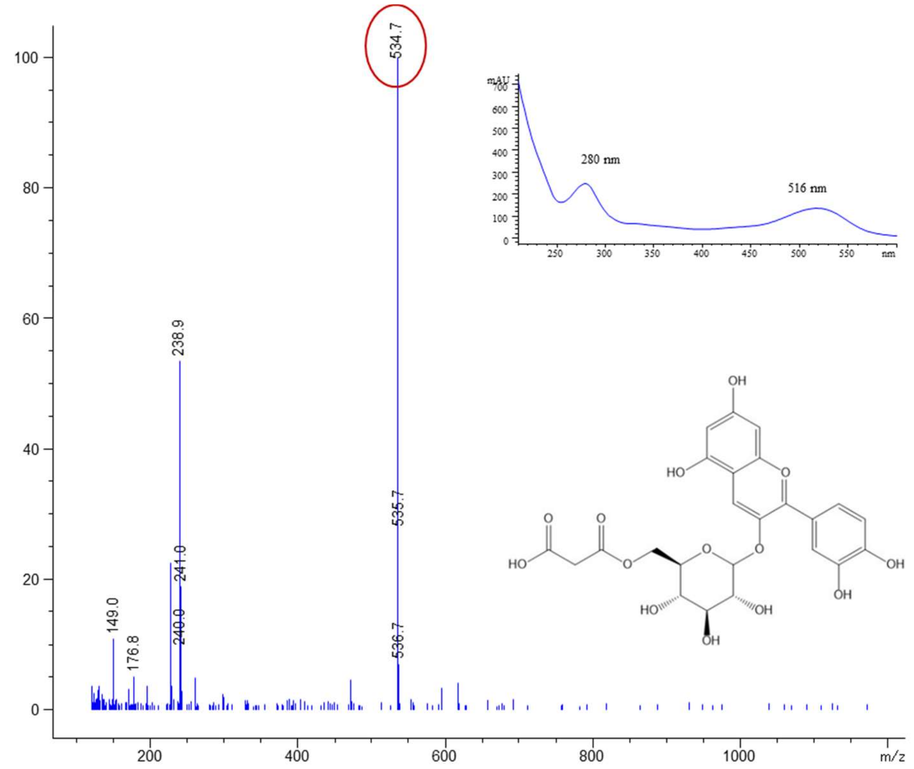

(h) peak 8
Cyanidin-3-*O*-(6″-malonyl-glucoside)

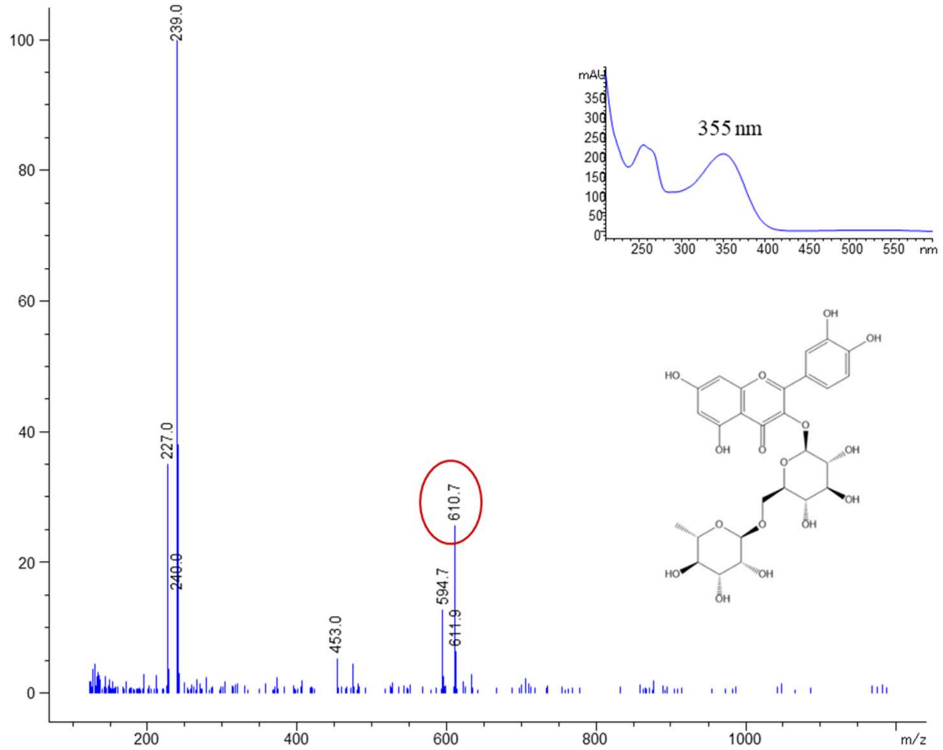

(i) peak 9
Quercetin-3-O-
rutinoside (Rutin)

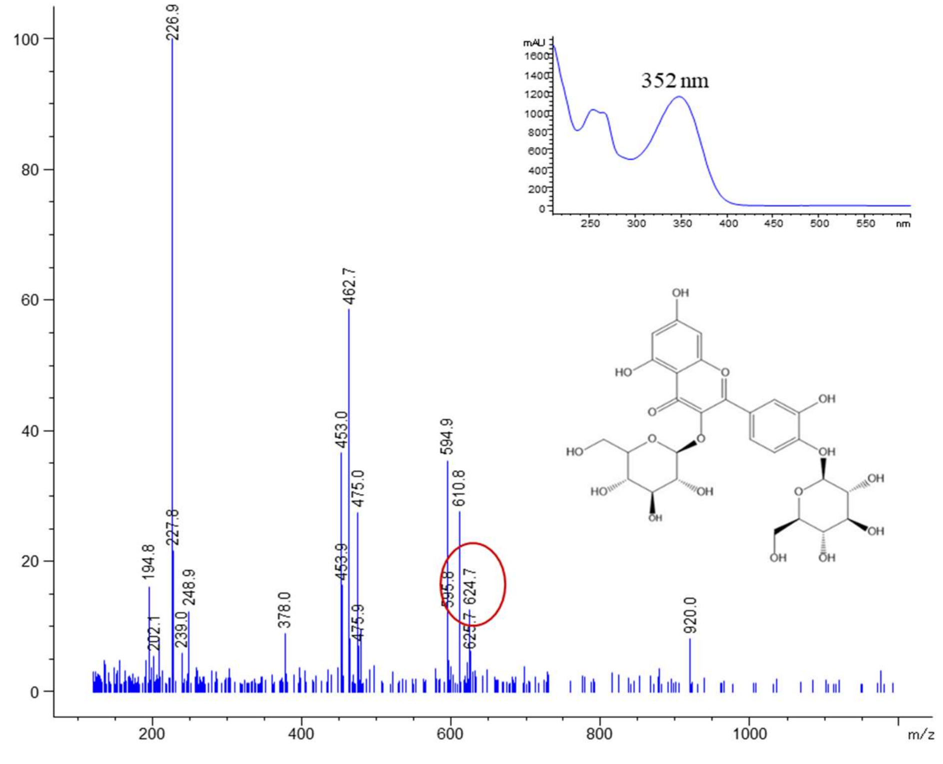

(j) peak 10
Quercetin-*3,4-O*-
diglucoside

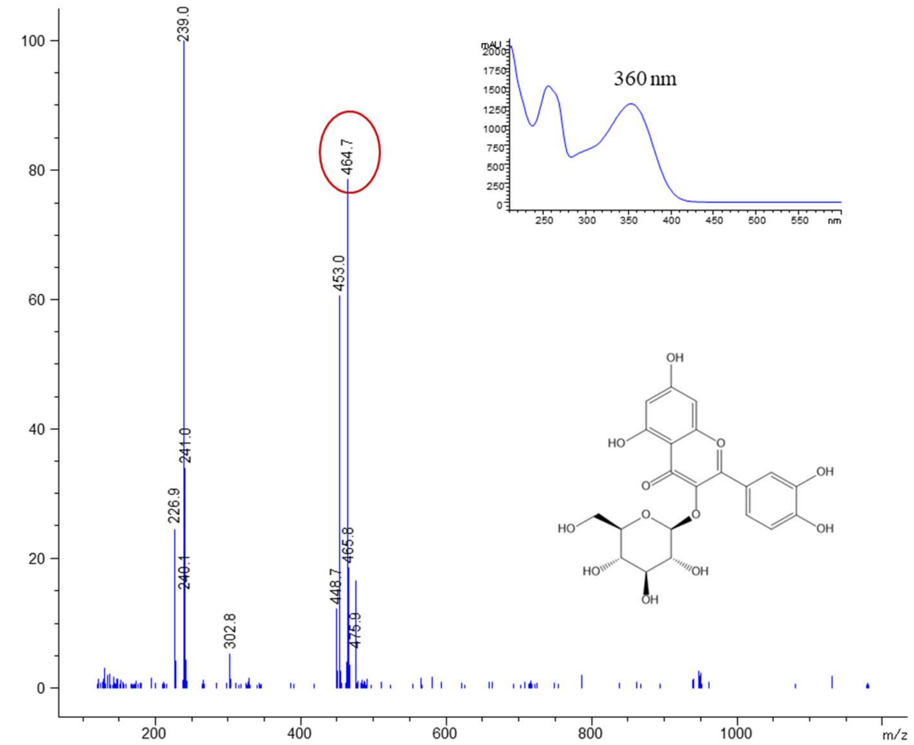

(k) peak 11
Quercetin-3-*O*-
glucoside

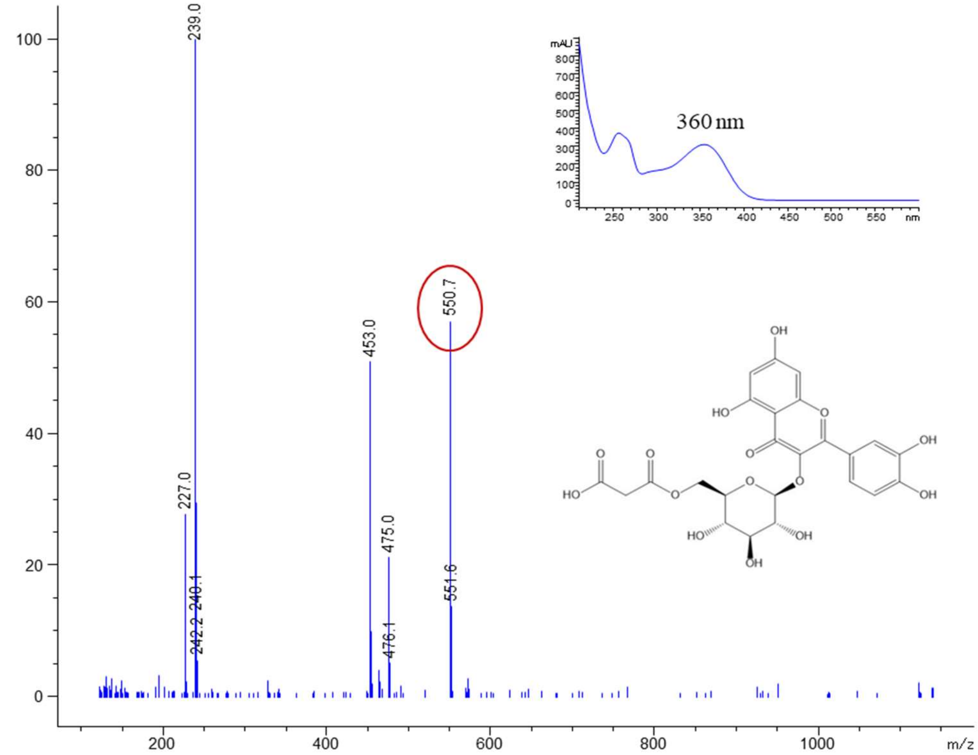

(l) peak 12
Quercetin-3-O-(6″-
malonyl-glucoside)

Figure 1. LC-MS; UV/vis scanning spectra; chemical structures of red chicory: (a) peak 1, (b) peak 2, (c) peak 3, (d) peak 4, (e) peak 5, (f) peak 6, (g) peak 7, (h) peak 8, (i) peak 9, (j) peak 10, (k) peak 11, (l) peak 12.

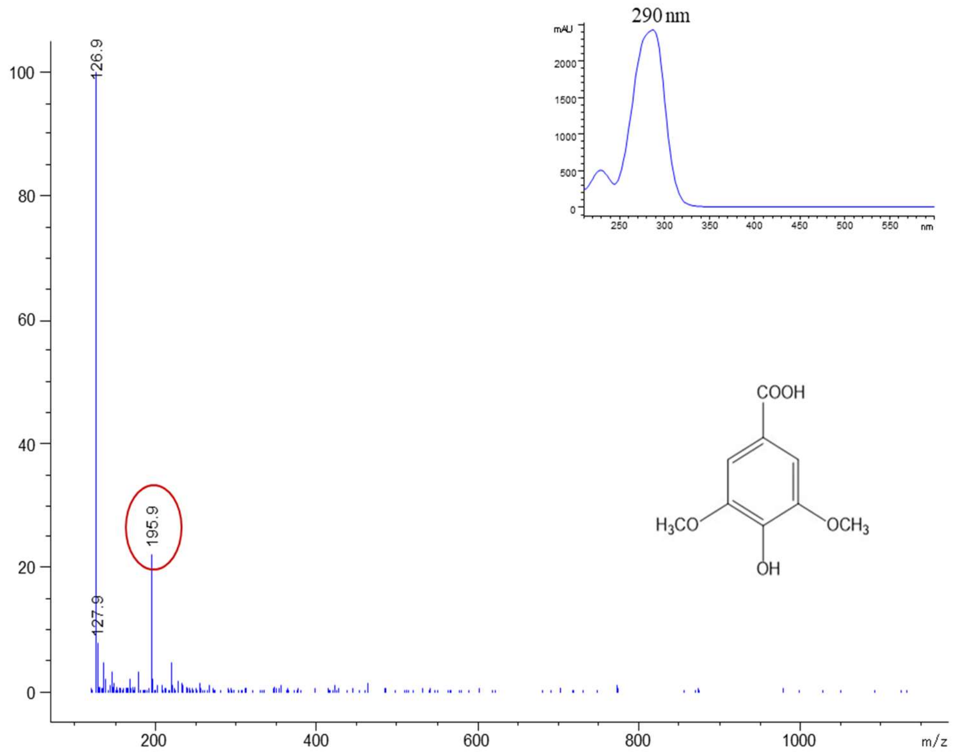

(a) peak 1
Syringic acid

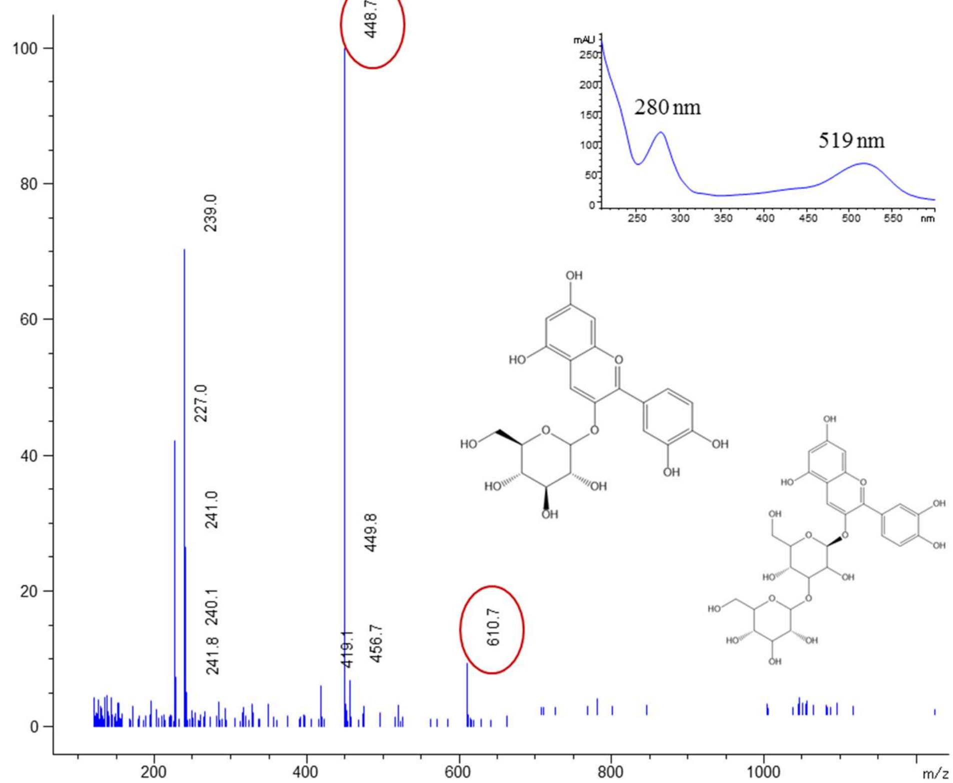

(b) peak 2
Cyanidin-*3*-*O*-glucoside + Cyanidin-*3*-*O*-laminaribioside

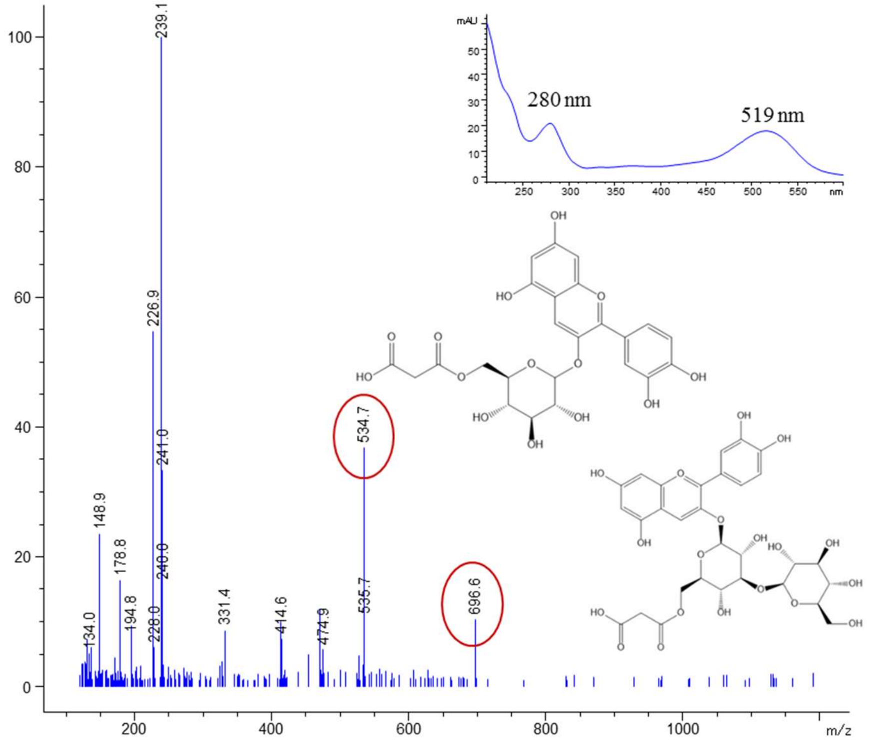

(c) peak 3 Cyanidin-3-(6″-malonyl-glucoside) + Cyanidin-3-(6″-malonyl-laminaribioside)

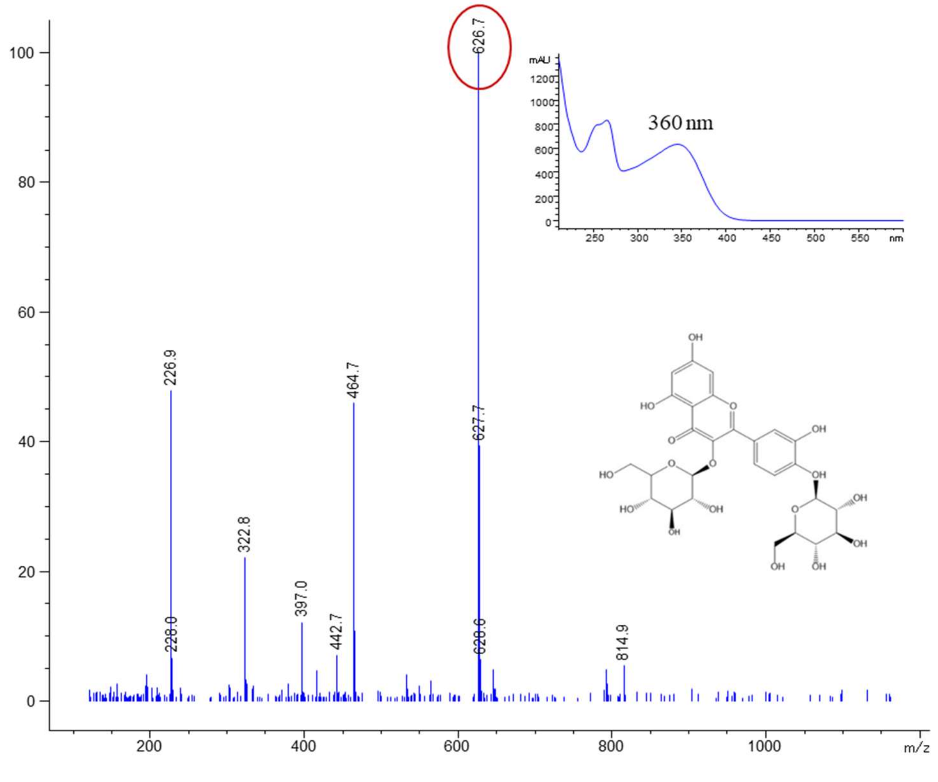

(d) peak 4 Quercetin-*3,4-O*-diglucoside

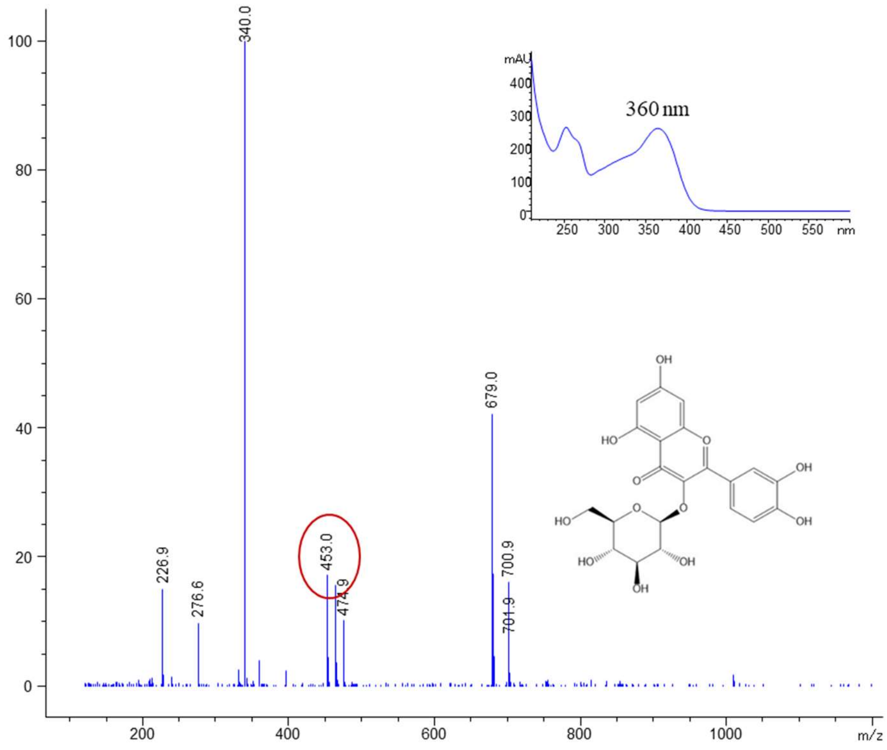

(e) peak 5
Quercetin-3-*O*-
glucoside

Figure 2. LC-MS; UV/VIS scanning spectra; chemical structures of red onion: (a) peak 1, (b) peak 2, (c) peak 3 (d) peak 4, (e) peak 5

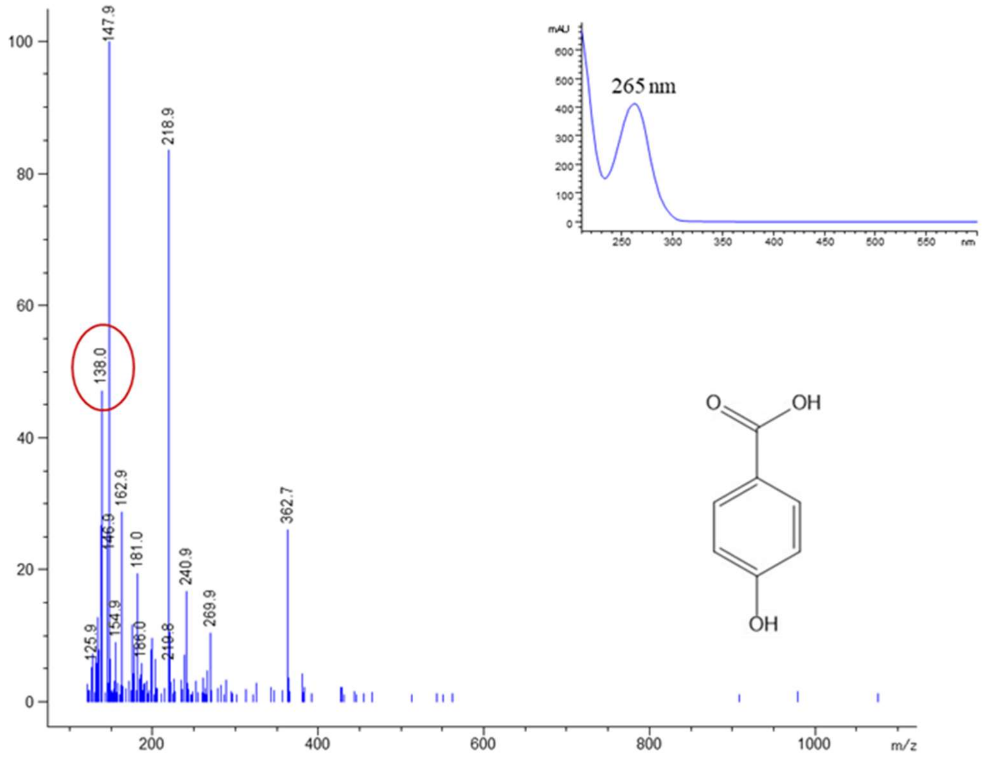

(a) peak 1
Hydroxybenzoic acid

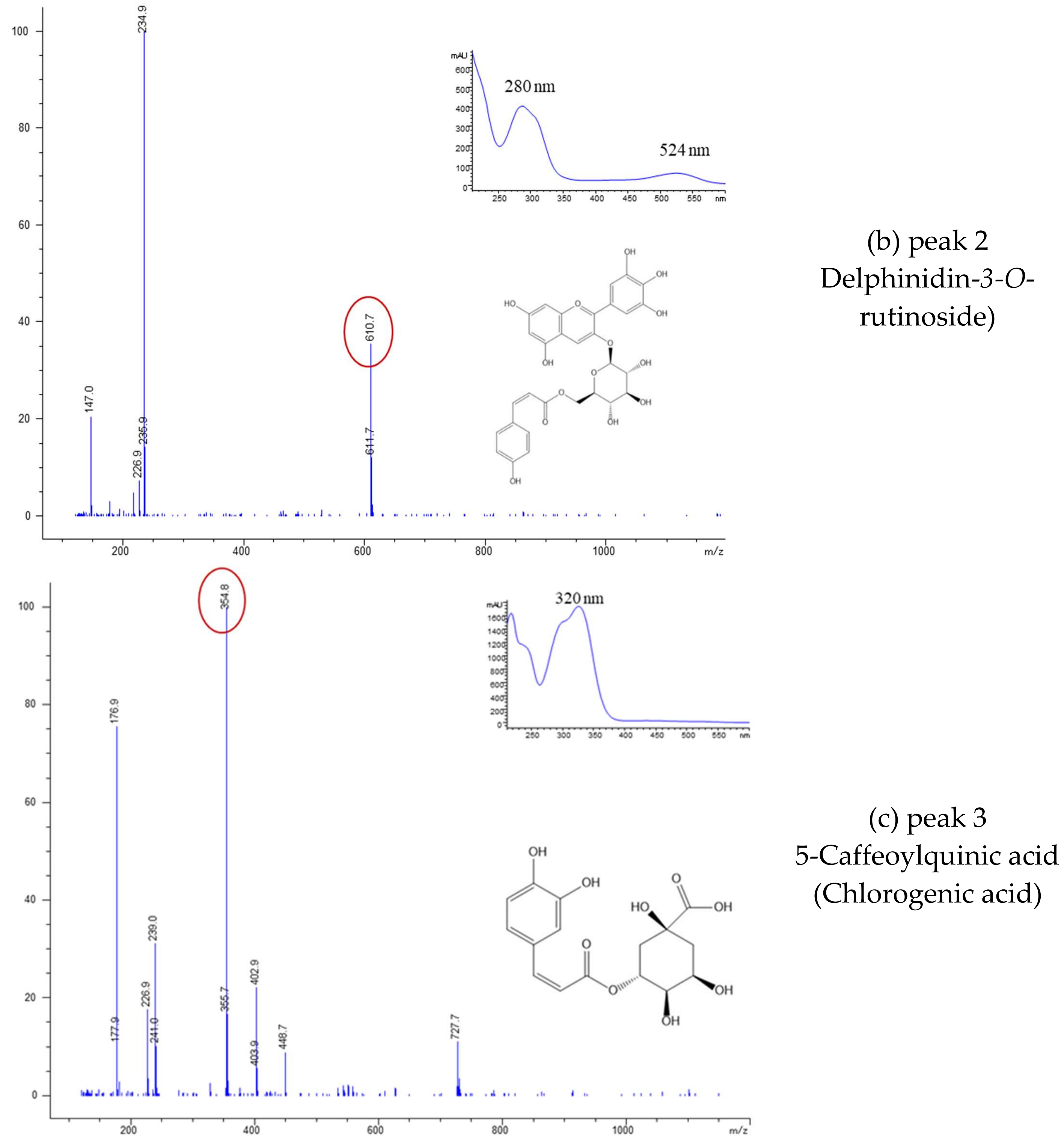

Figure 3. LC-MS; UV/vis scanning spectra; chemical structures of eggplant: (a) peak 1, (b) peak 2, (c) peak 3.

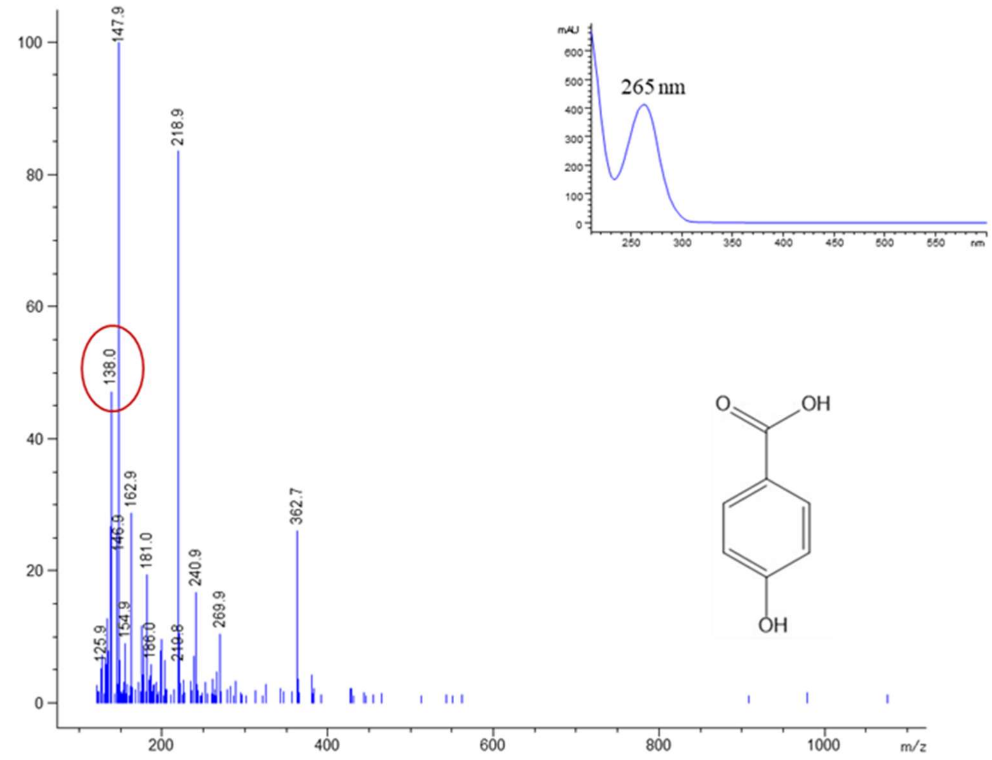

(a) peak 1

Hydroxybenzoic acid

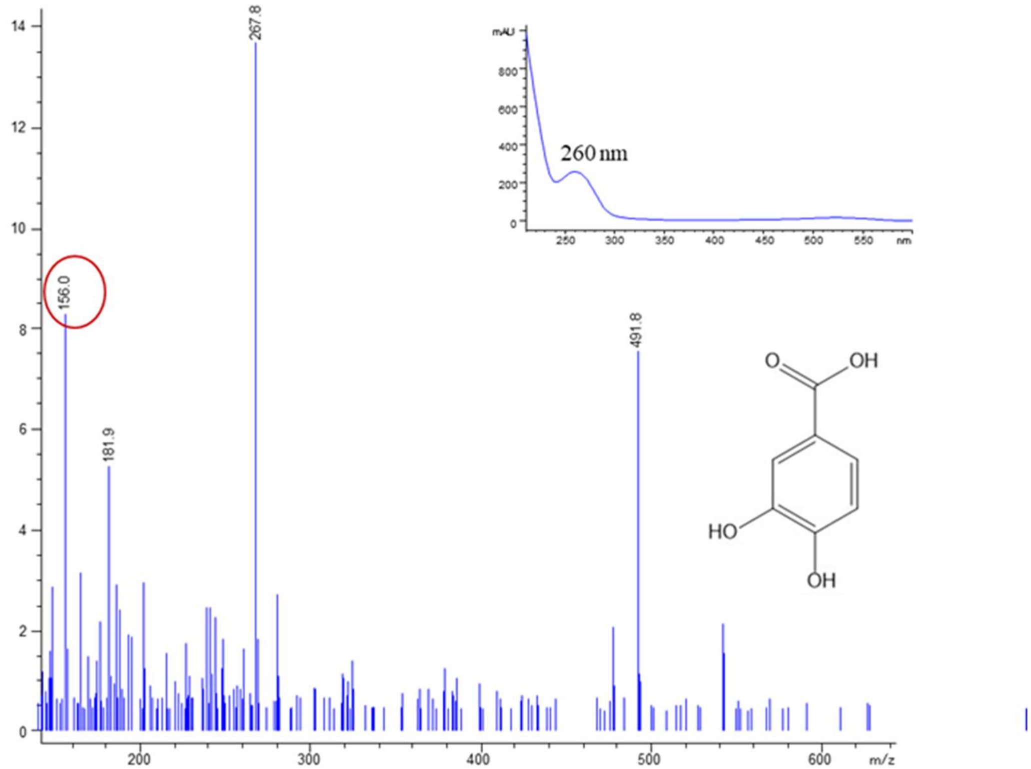

(b) peak 2
Dihydroxybenzoic acid

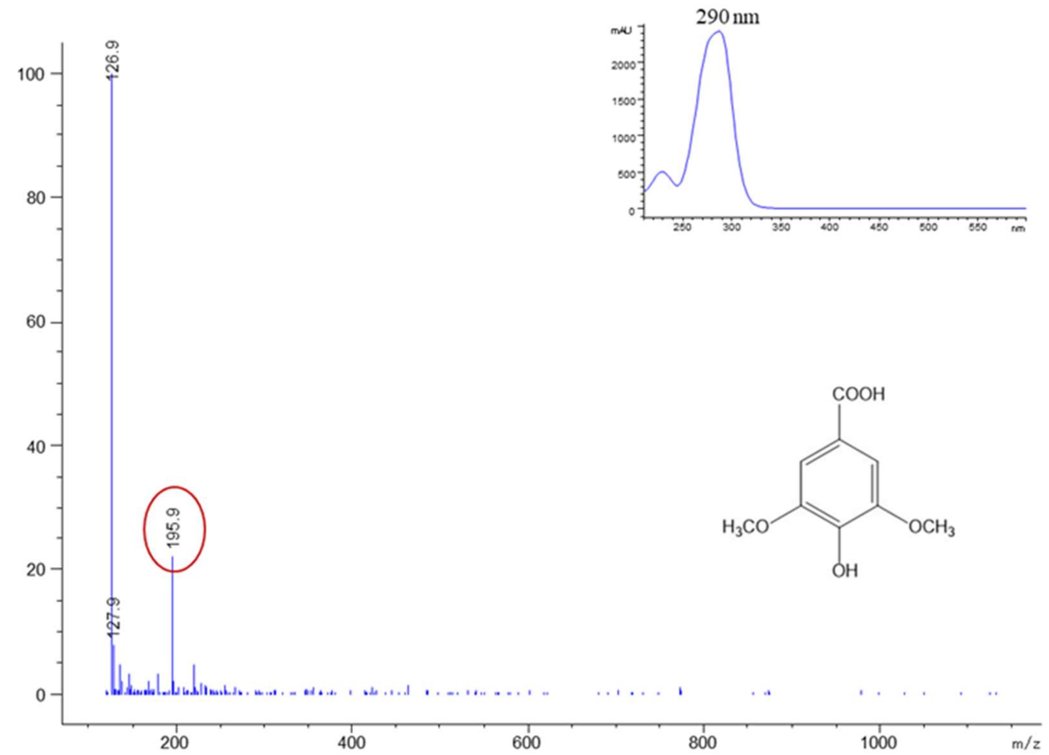

(c) peak 3
Syringic acid

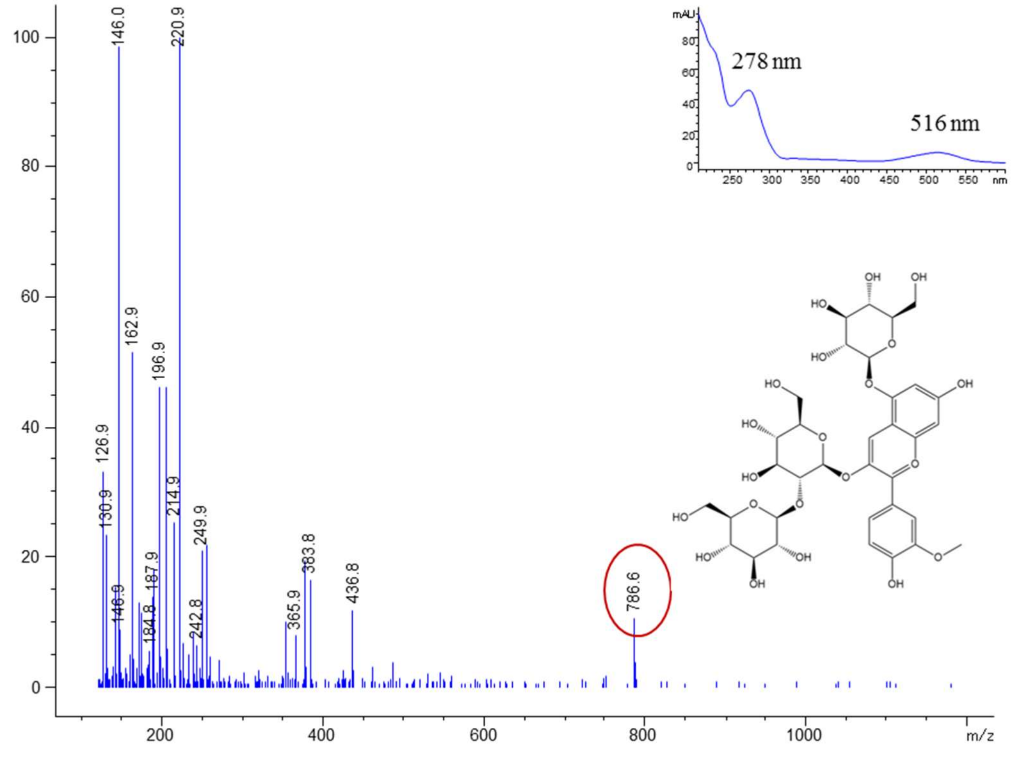

(d) peak 4
Peonidin-3-*O*-sophoroside-5-*O*-glucoside

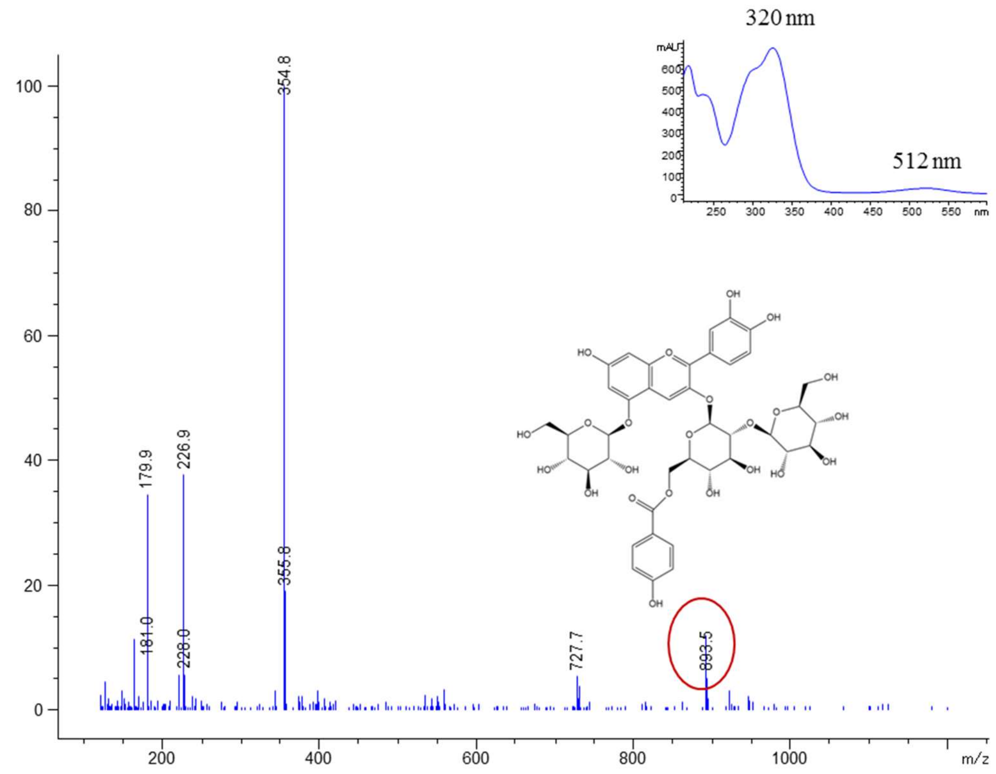

(e) peak 5
Peonidin-*3*-*O*-
glucoside

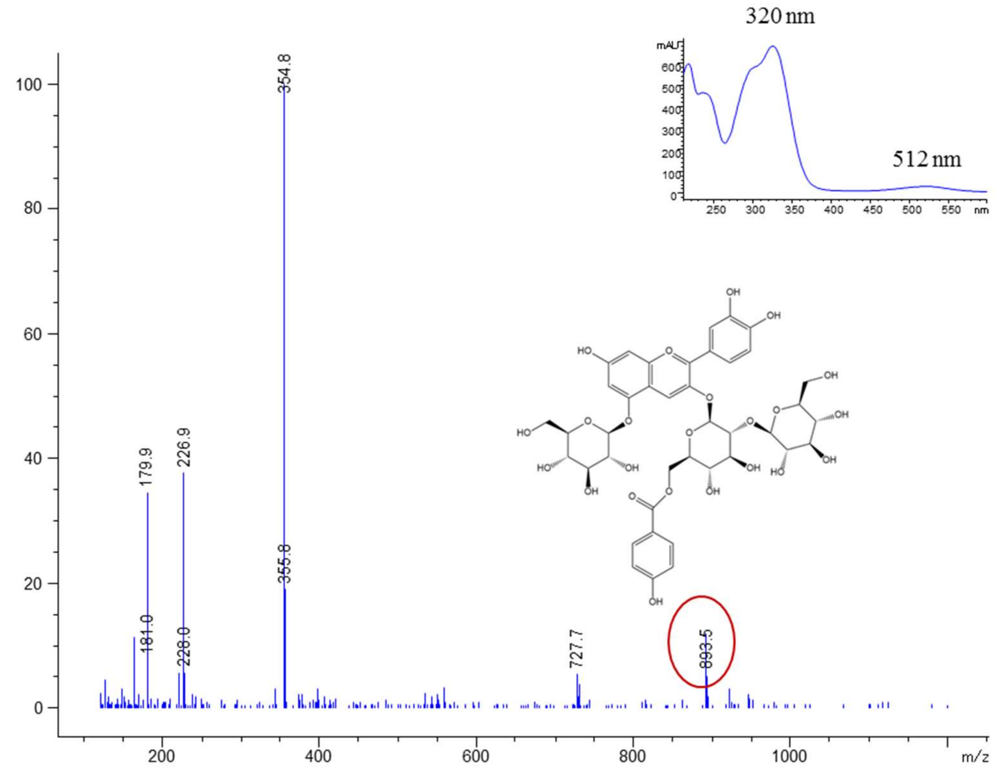

(f) peak 6
Cyanidin-3-p-
hydroxybenzoyl
sophoroside-5-
glucoside

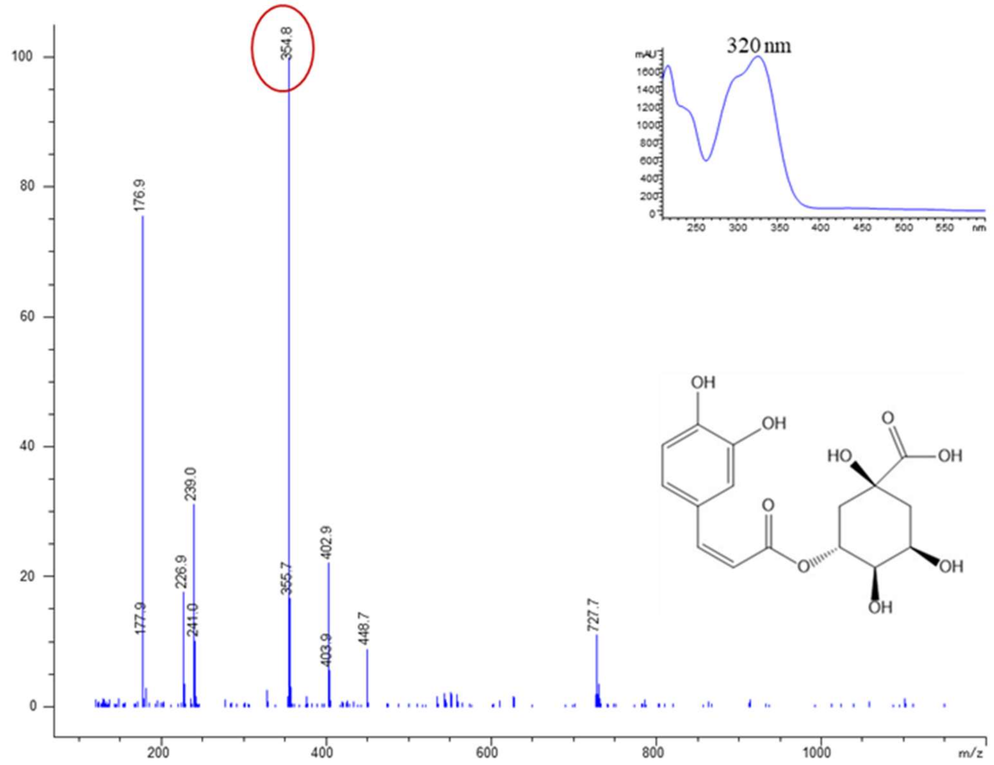

(g) peak 7
Chlorogenic acid

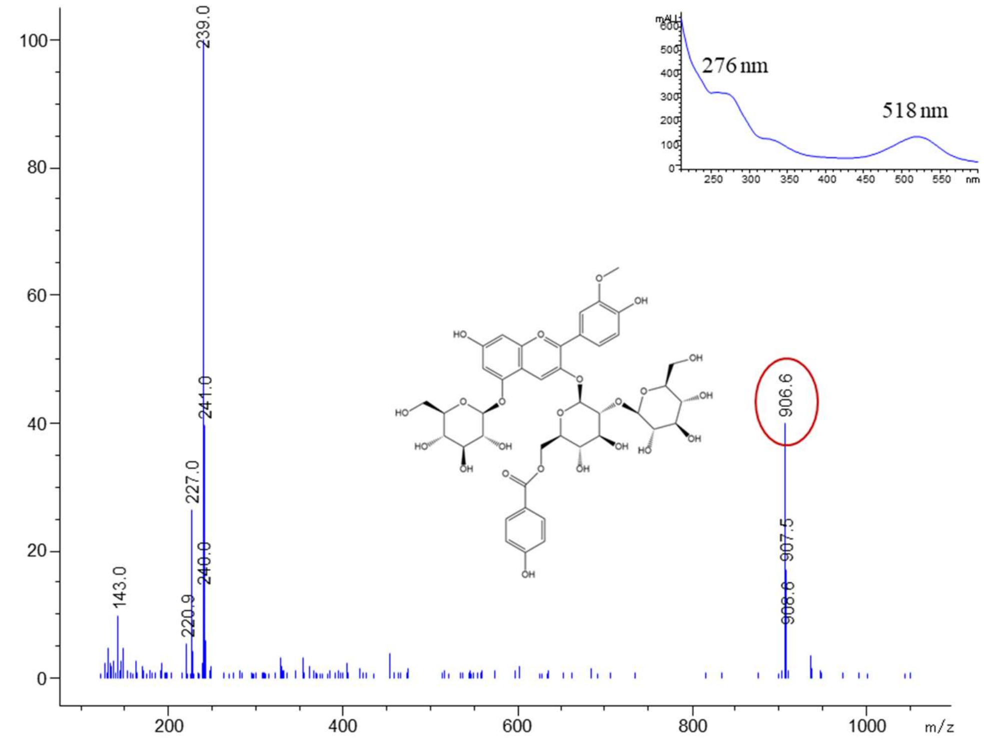

(h) peak 8
Peonidin-3-p-hydroxybenzoyl sophoroside-5-glucoside

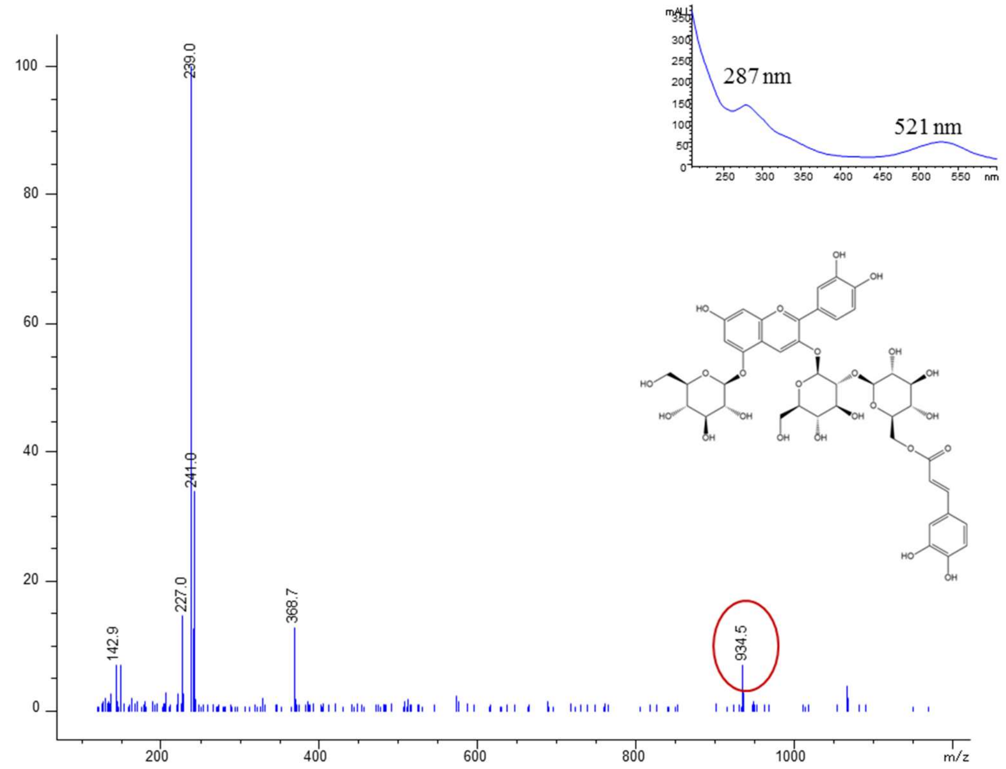

(i) peak 9
Cyanidin-3-caffeoylsophoroside-5-glucoside

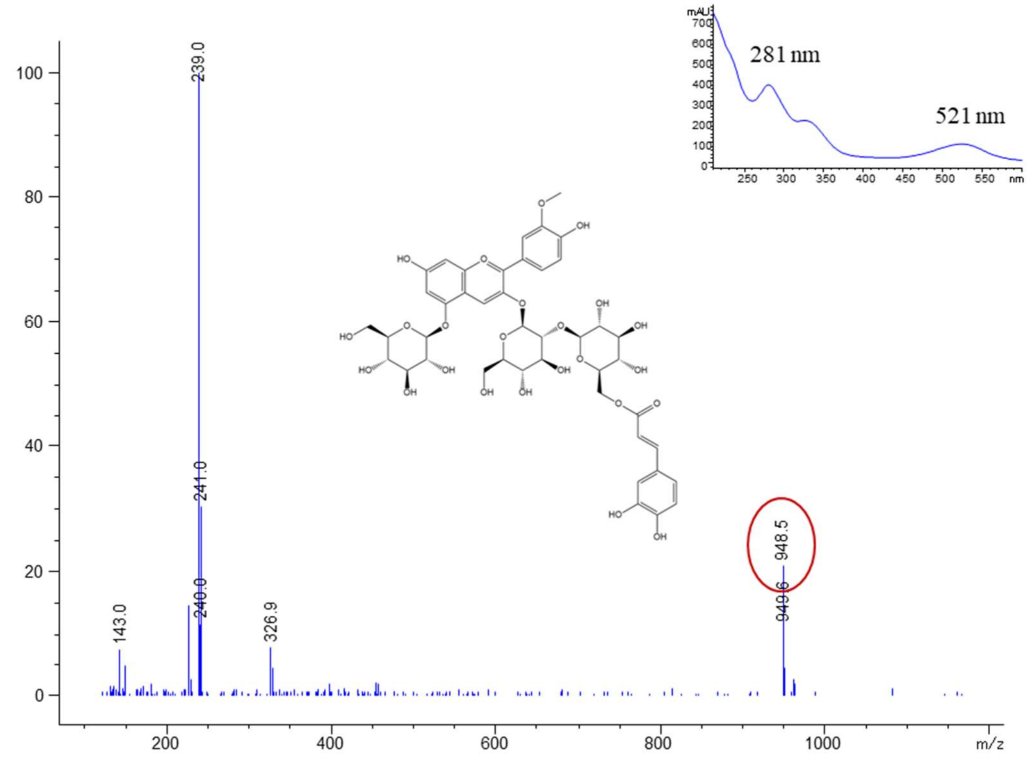

(j) peak 10
Peonidin-3-caffeoylsophoroside-5-glucoside

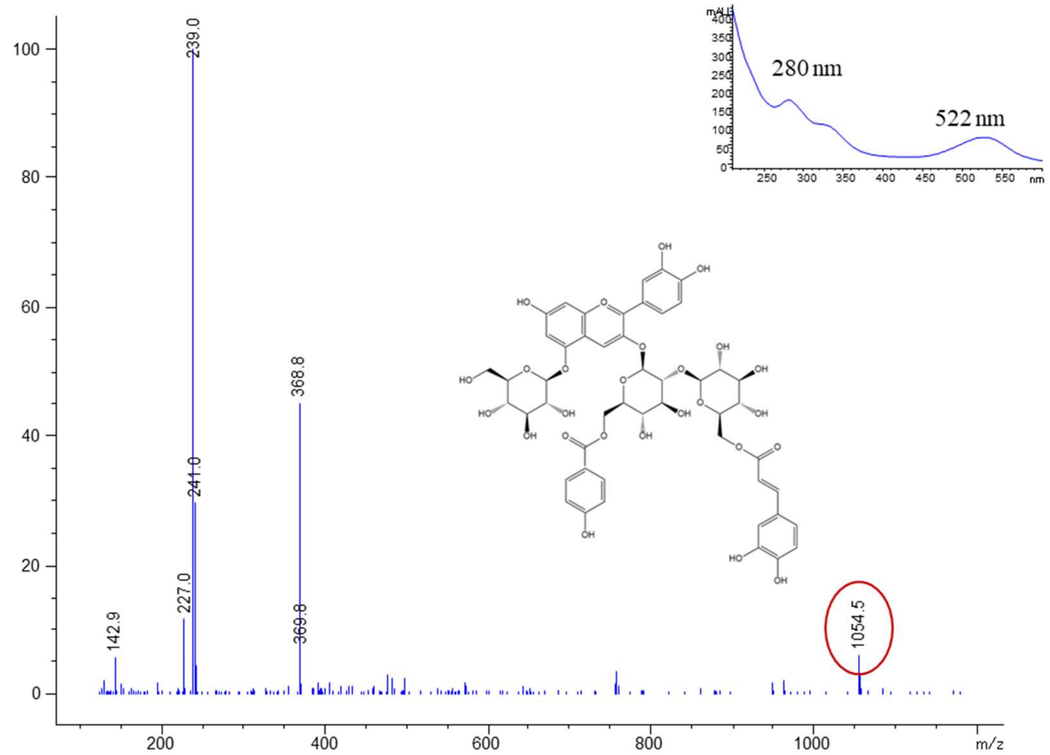

(k) peak 11
Cyanidin-3-caffeoyl-p-hydroxybenzoyl sophoroside-5-glucoside

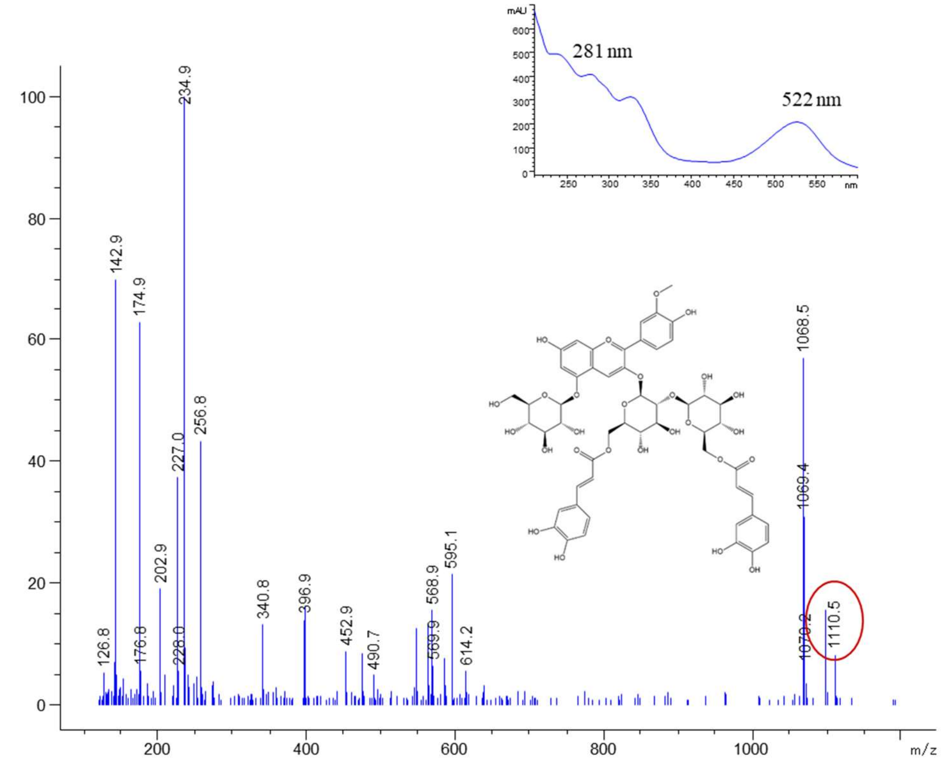

(l) peak 12
Peonidin-3-dicaffeoylsophoroside-5-glucoside

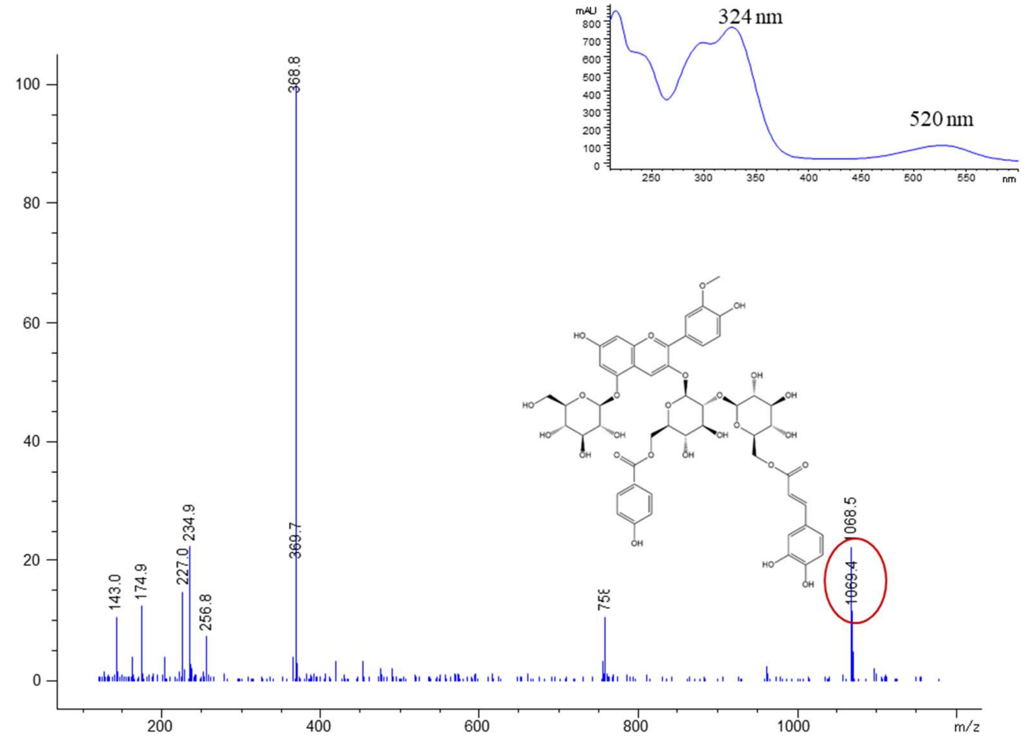

(m) peak 12 Peonidin-3-caffeoyl-p-hydroxybenzoyl sophoroside-5-glucoside

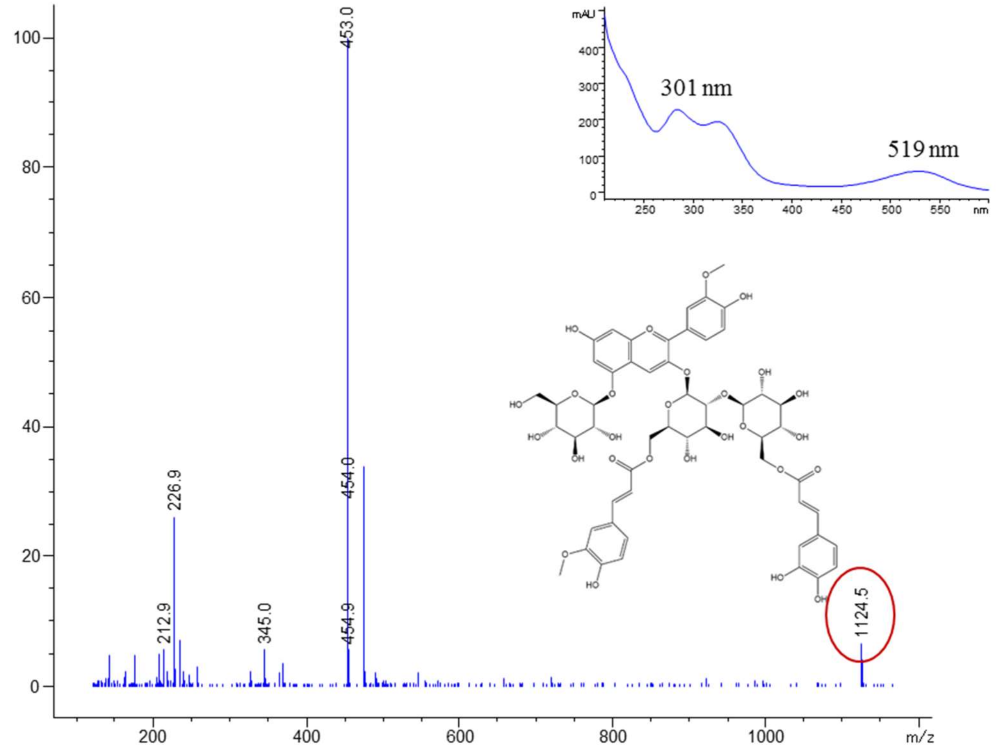

(n) peak 13 Peonidin-3-caffeoy-feruloylsophoroside-5-glucoside

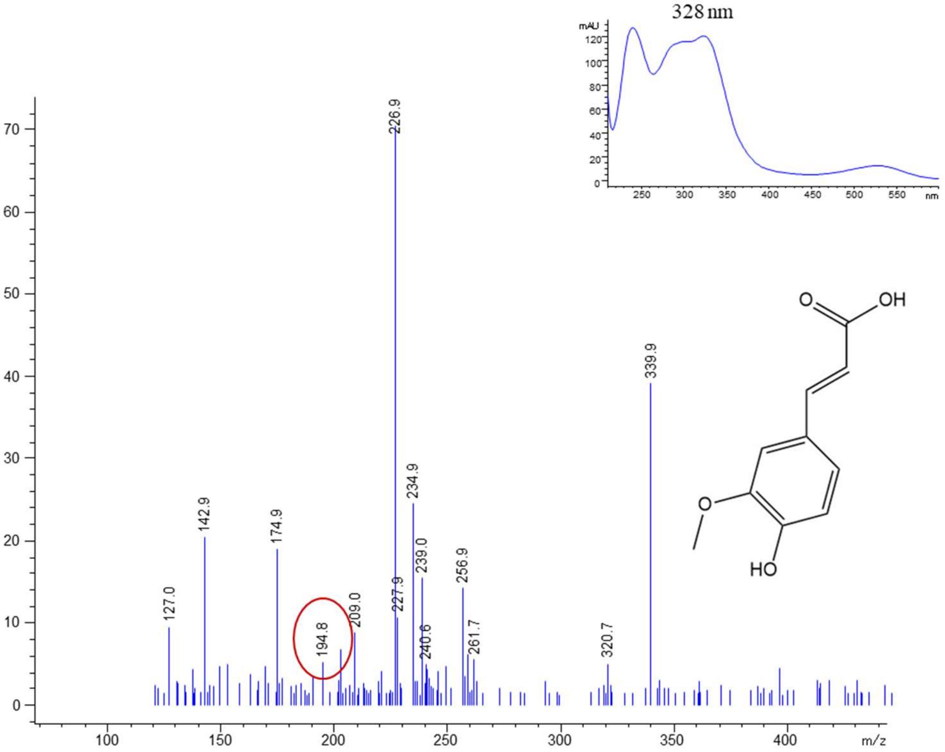

(o) peak 14
Ferulic acid

Figure 4. LC-MS; UV/vis scanning spectra; chemical structures of purple sweet potato: (a) peak 1, (b) peak 2, (c) peak 3, (d) peak 4, (e) peak 5, (f) peak 6, (g) peak 7, (h) peak 8, (i) peak 9, (j) peak 10, (k) peak 11, (l) peak 12, (m) peak 12, (n) peak 13,(o) peak 14.

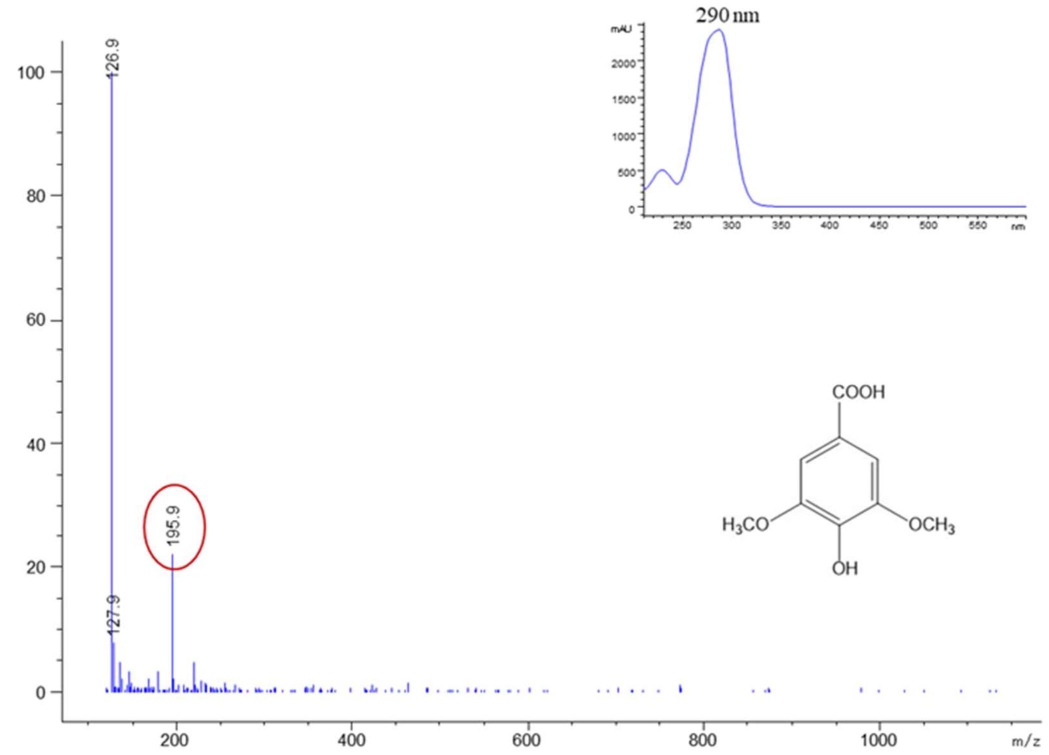

(a) peak 1
Syringic acid

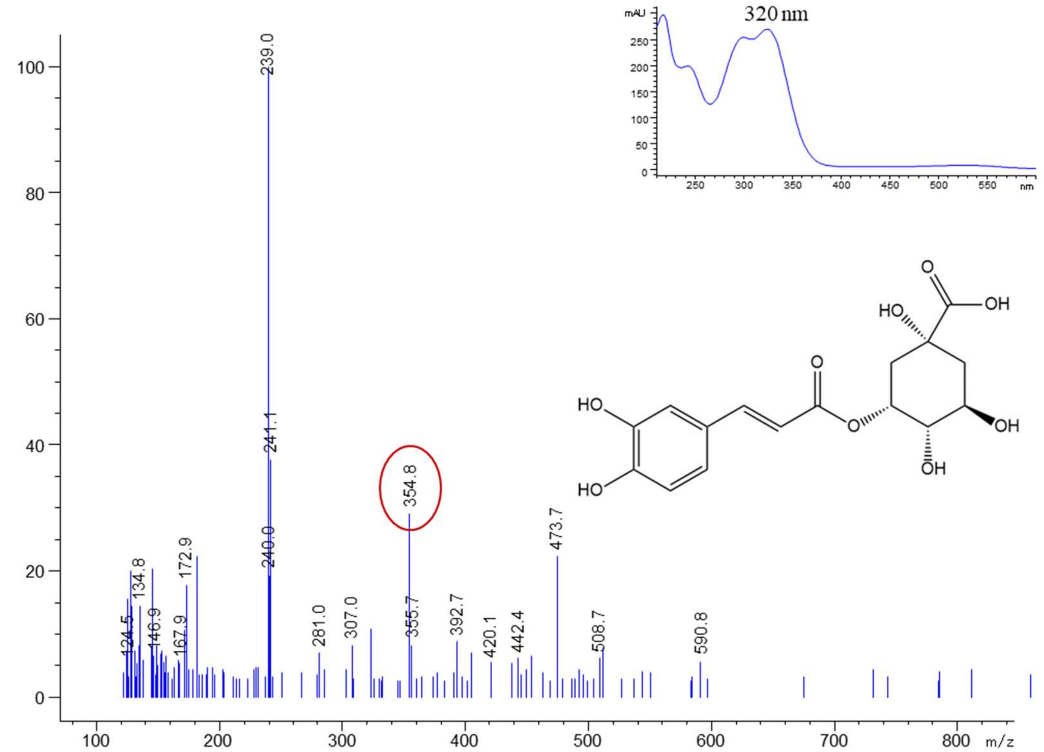

(b) peak 2
3-Caffeoylquinic
acid
(Neochlorogenic
acid)

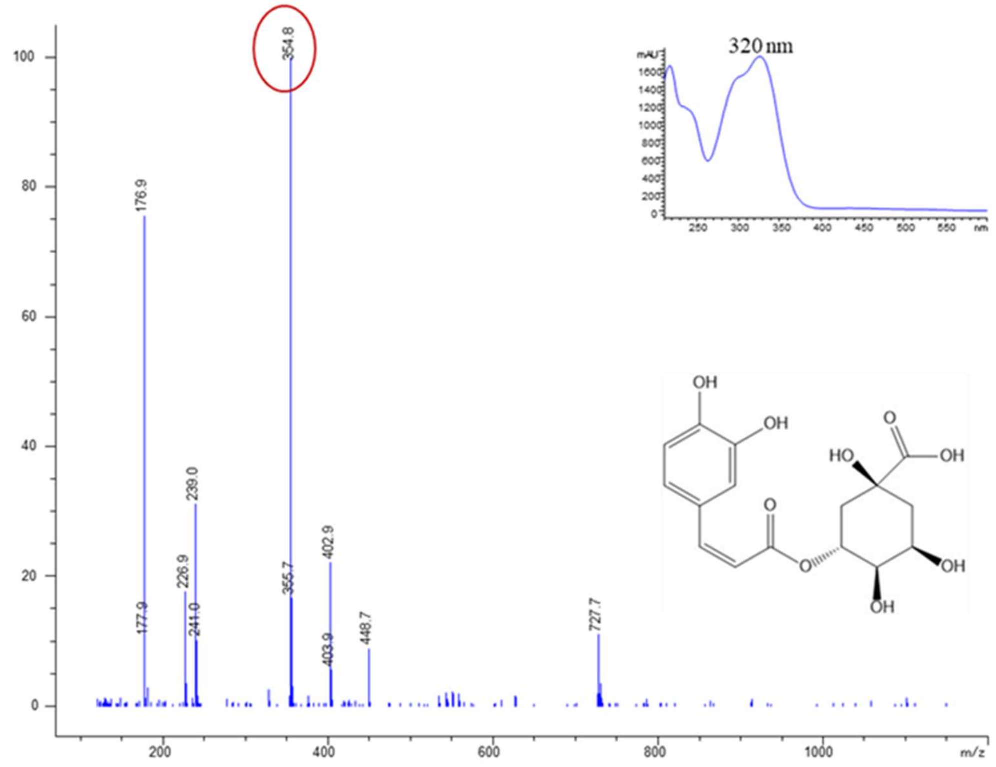

(c) peak 3
5-Caffeoylquinic acid (Chlorogenic acid)

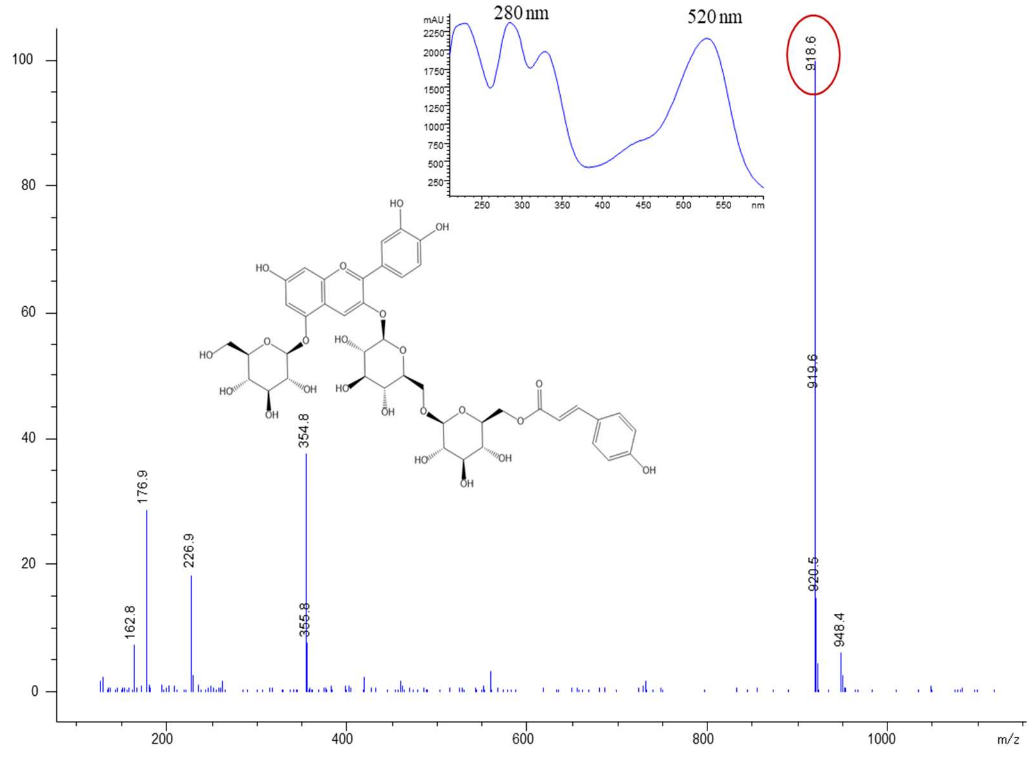

(d) peak 3
Cyanidin-3-(p-coumaroyl)-diglucoside-5-glucoside

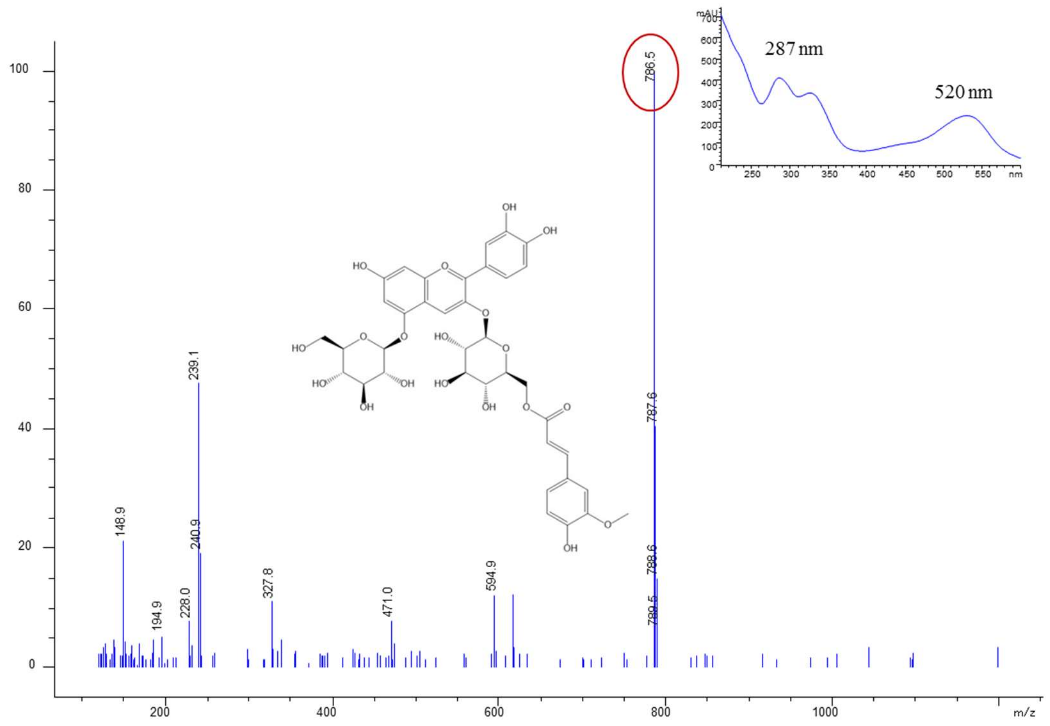

(e) peak 4
Cyanidin-3-(feruloyl)-glucoside-5-glucoside

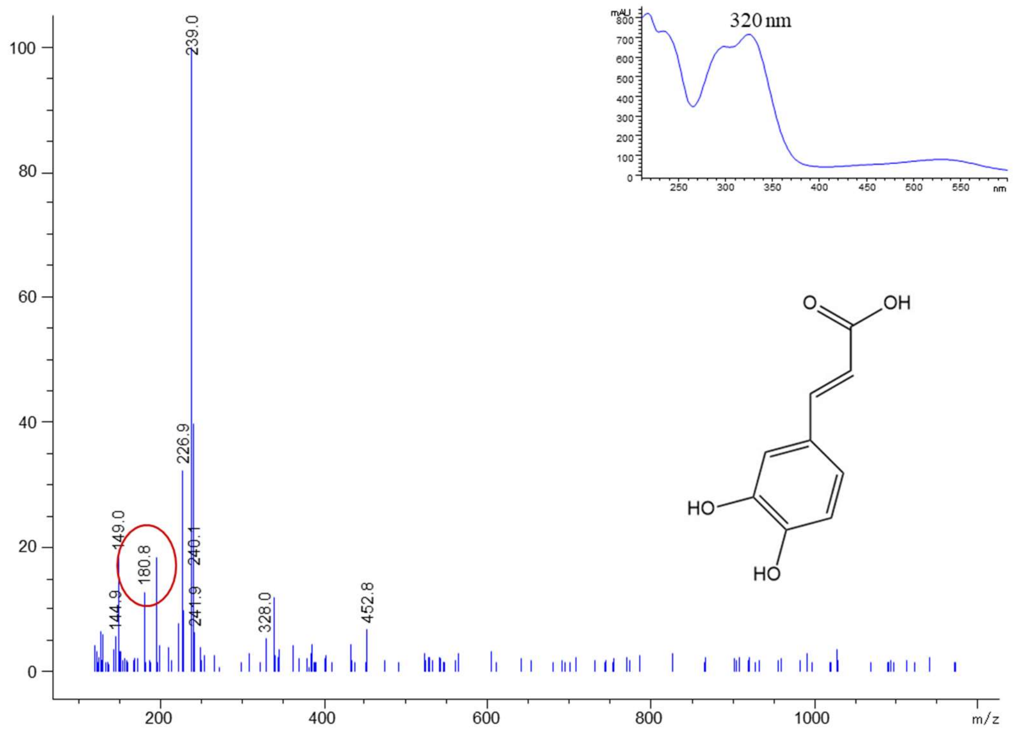

(f) peak 5
Caffeic acid

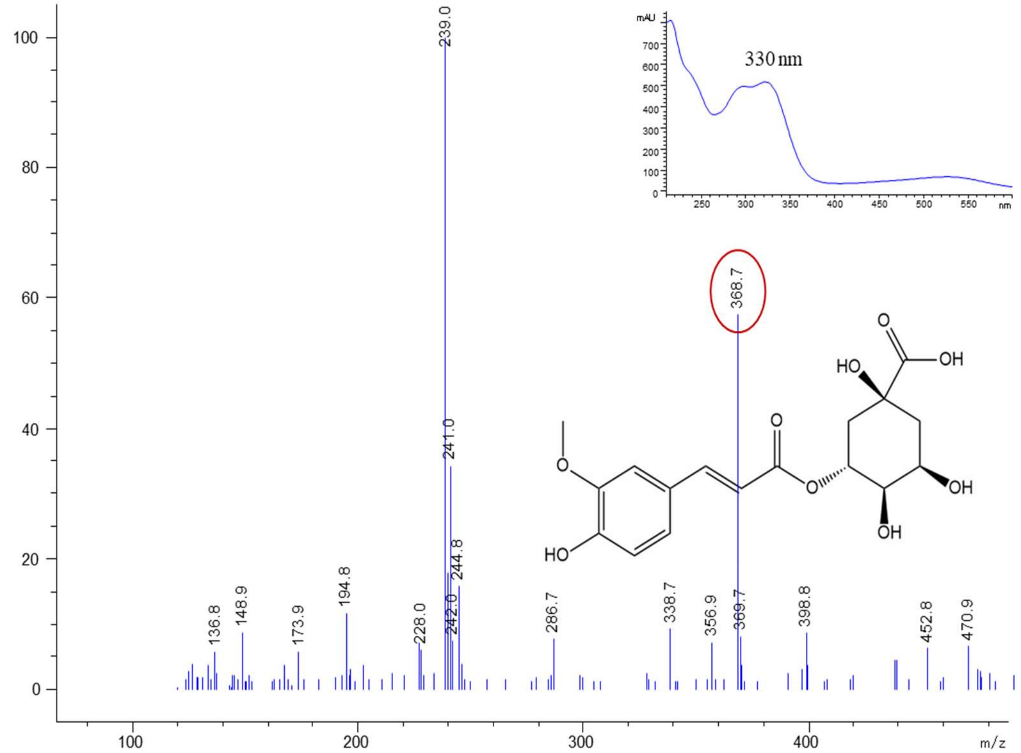

(g) peak 6
Feruloylquinic acid

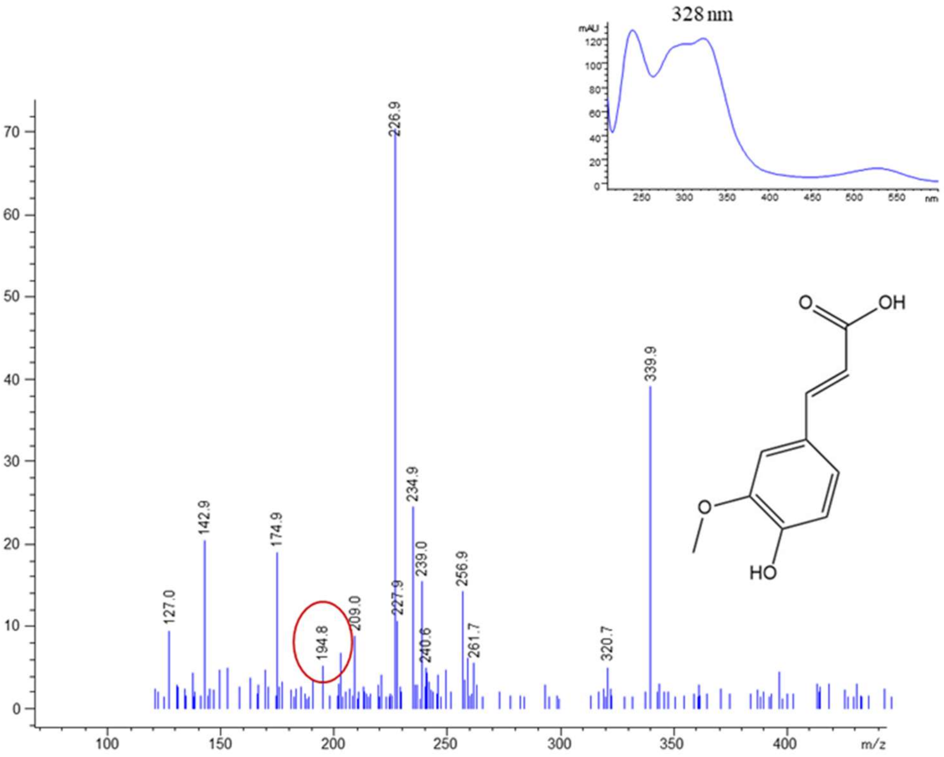

(h) peak 7
Ferulic acid

Figure 5. LC-MS; UV/VIS scanning spectra; chemical structures of black carrot: (a) peak 1, (b) peak 2, (c) peak 3, (d) peak 4, (e) peak 5, (f) peak 6, (g) peak 7, (h) peak 8.
